# Microgrewiapine C: Asymmetric Synthesis, Spectroscopic Data, and Configuration Assignment

**DOI:** 10.1021/acs.jnatprod.2c00183

**Published:** 2022-06-30

**Authors:** Stephen G. Davies, Ai M. Fletcher, Paul M. Roberts, Cameron E. Taylor, James E. Thomson

**Affiliations:** Department of Chemistry, Chemistry Research Laboratory, University of Oxford, Mansfield Road, Oxford OX1 3TA, U.K.

## Abstract

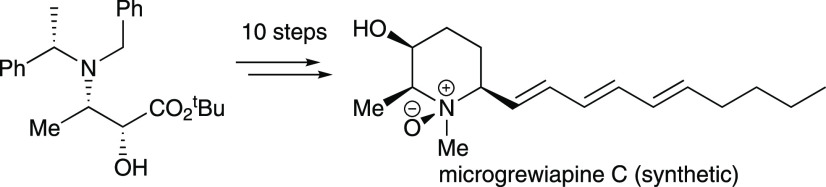

The first asymmetric synthesis of microgrewiapine C, a piperidine alkaloid isolated from *Microcos paniculata*, is reported. This synthesis prompted correction of the ^1^H and ^13^C NMR data for the natural sample of the alkaloid, which was achieved by reanalysis of the original spectra. The corrected data for the natural product were found to be identical to those of the synthetic sample prepared herein, thus confirming the structural and relative configurational assignment of microgrewiapine C. Although comparison of specific rotation values indicates that the (1*R*,2*S*,3*S*,6*S*) absolute configuration should be assigned to the alkaloid, consideration of potential common biosynthetic origins of microgrewiapine C and congeners suggests that further phytochemical investigations are warranted.

Kinghorn et al. reported the results of their phytochemical investigations of various parts of the shrubby tree *Microcos paniculata* in 2013.^1^ The same extraction procedure was applied to the stem bark, branches, and leaves of the plant and revealed a different alkaloidal content: microgrewiapine A was isolated from the stem bark, microgrewiapine B and microgrewiapine C were isolated from the branches, and microcosamine A was isolated from the leaves (Figure 1).^1^ These observations suggest that all four are genuine secondary metabolites produced by *M. paniculata*, i.e., that the *N*-oxides microgrewiapine B and microgrewiapine C are not merely artifacts of the extraction process (produced on aerial *N*-oxidation of the parent compounds). As such, they all belong to a growing family of alkaloids based on a 2-methyl-6-(deca-1′,3′,5′-trienyl)piperidine-3-ol core that have been isolated from this organism.^1−6^ Most of these alkaloids have been assigned the 2,3-*trans*-3,6-*trans* relative configuration,^1,3−5^ as illustrated by the structures of microgrewiapine A,^1^ microgrewiapine B,^1^ and microcosamine A^3^ (Figure 1), meaning that the 2,3-*cis*-3,6-*cis* relative configuration assigned to microgrewiapine C^1^ is somewhat unusual: in fact, only one other alkaloid originating from *M. paniculata* has thus far been found to possess the 2,3-*cis*-3,6-*cis* relative configuration, viz., microconine^2^ (Figure 1). Although only the relative configurations of both microgrewiapine C^1^ and microconine^2^ were assigned in the original isolation studies (chiefly by ^1^H NMR ^3^*J* coupling constant analysis), our recent total synthesis of microconine^7^ resulted in the (2*R*,3*R*,6*R*) absolute configuration being proposed for this alkaloid upon comparison of the specific rotation of our synthetic sample of known (2*S*,3*S*,6*S*) absolute configuration {[α]_D_^22^ −29.0 (*c* 1.0, CHCl_3_)}^7^ with that of the natural sample {[α]_D_^22^ +29.2 (CHCl_3_)}.^2,8^ Meanwhile, Kinghorn et al. performed a Mosher’s ester analysis on microgrewiapine A and on this basis assigned it the (2*S*,3*R*,6*S*) absolute configuration.^1^ It was then speculated that microgrewiapine B, which possessed the same relative configuration as microgrewiapine A of the three common stereogenic centers around the piperidine ring, most likely shared the (2*S*,3*R*,6*S*) absolute configuration of these stereogenic centers and thus the (1*R*)-(2*S*,3*R*,6*S*) absolute configuration was assigned to microgrewiapine B. Meanwhile, it was assumed that microgrewiapine C possessed the (2*S*,3*S*,6*S*) absolute configuration of the three common stereocenters on the basis that it was most simply related to microgrewiapine A and microgrewiapine B as the C-3 epimer (although no direct evidence to substantiate this assignment was presented). Hence, the (1*R*)-(2*S*,3*S*,6*S*) absolute configuration was assigned to microgrewiapine C.^1^

**Figure 1:**
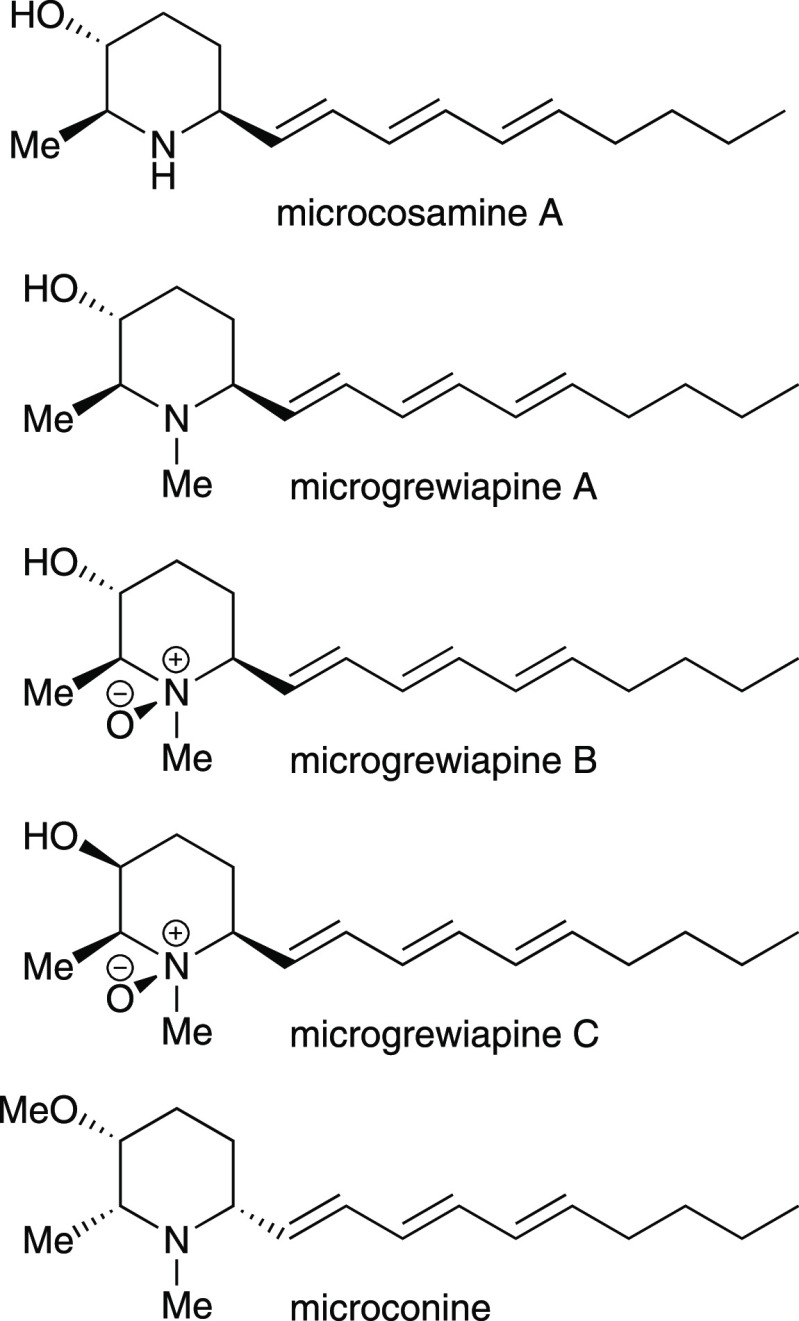
Structures of microcosamine A, microgrewiapine A, microgrewiapine B, microgrewiapine C, and microconine. Absolute configurations shown are those assigned in the original isolation studies (microgrewiapine A, microgrewiapine B, microgrewiapine C) and/or those confirmed/established by total synthesis (microcosamine A, microgrewiapine A, microgrewiapine B, and microconine).

We have initiated a research program to synthesize various members of this alkaloid family so that their structural and absolute configuration assignments can be investigated,^7,9,10^ and have already described the preparation of a number of these alkaloids (*viz*., microconine,^7^ microcosamine A,^9^ microgrewiapine A,^9^ and microgrewiapine B).^9^ It was resolved to extend these synthetic endeavors to encompass microgrewiapine C and so allow its relative and absolute configuration assignments to be investigated. The results of these studies are reported herein and comprise the execution of the first asymmetric synthesis of this alkaloid.

The synthesis of microgrewiapine C drew on our previous experience concerning the syntheses of these alkaloids^7,9^ and commenced with the known enantiopure *syn*-α-hydroxy-β-amino ester **1**, which is of unambiguously established (2*R*,3*S*,α*S*) absolute configuration^7,11,12^ and which we have previously elaborated to the (2*S*,3*S*,6*S*)-enantiomer of microconine.^7^ Treatment of **1** with NaH followed by MOMCl gave α-methoxymethyloxy-β-amino ester **2** in 99% yield. Subsequent reduction of **2** using DIBAL-H gave the corresponding aldehyde **3** directly, enabling Wittig-type olefination using Ph_3_P=CHCO_2_Et to give α,β-unsaturated ester **4** as a single diastereoisomer (>95:5 dr [(*E*):(*Z*) ratio]) in 81% isolated yield from **2**. Treatment of **4** with Boc_2_O in the presence of Pd(OH)_2_/C under a hydrogen atmosphere effected tandem reduction of the olefin, *N*-debenzylation, and *N*-Boc protection, furnishing **5** in 85% yield. Elaboration of **5** upon treatment with the lithium anion of MeP(O)(OMe)_2_ (formed in situ upon treatment with ^n^BuLi) gave β-keto phosphonate **6** in 92% yield. Wadsworth–Emmons-type olefination of (*E*,*E*)-2,4-nonadienal^13^ using β-keto phosphonate **6** gave triene **7**, which was treated as an intermediate and subjected to the sequential actions of TFA and then NaBH_3_CN (to effect removal of the *N*-Boc group followed by intramolecular reductive amination), which resulted in formation of **8** (3-*epi*-microcosamine A) in 17% isolated yield from **6**. Meanwhile, direct treatment of **8** (without purification) with formalin and NaBH_3_CN effected reductive *N*-methylation to give **9** (3-*epi*-microgrewiapine A) in 25% isolated yield from **6** after chromatography. Treatment of **9** with *m*-CPBA for 30 s effected *N*-oxidation to give **10** (3-*epi*-microgrewiapine B, synonymous with microgrewiapine C in the enantiomeric series assigned by Kinghorn et al.)^1^ in 47% isolated yield. As with our previous experience with these structures, the triene-containing compounds **8**–**10** were particularly sensitive to handling and purification (which accounts for the modest isolated yields), and some residual aliphatic impurities were evident in the sample in each case, although these did not prove problematic for the subsequent structural and stereochemical investigations (Scheme 1).

**Scheme 1:**
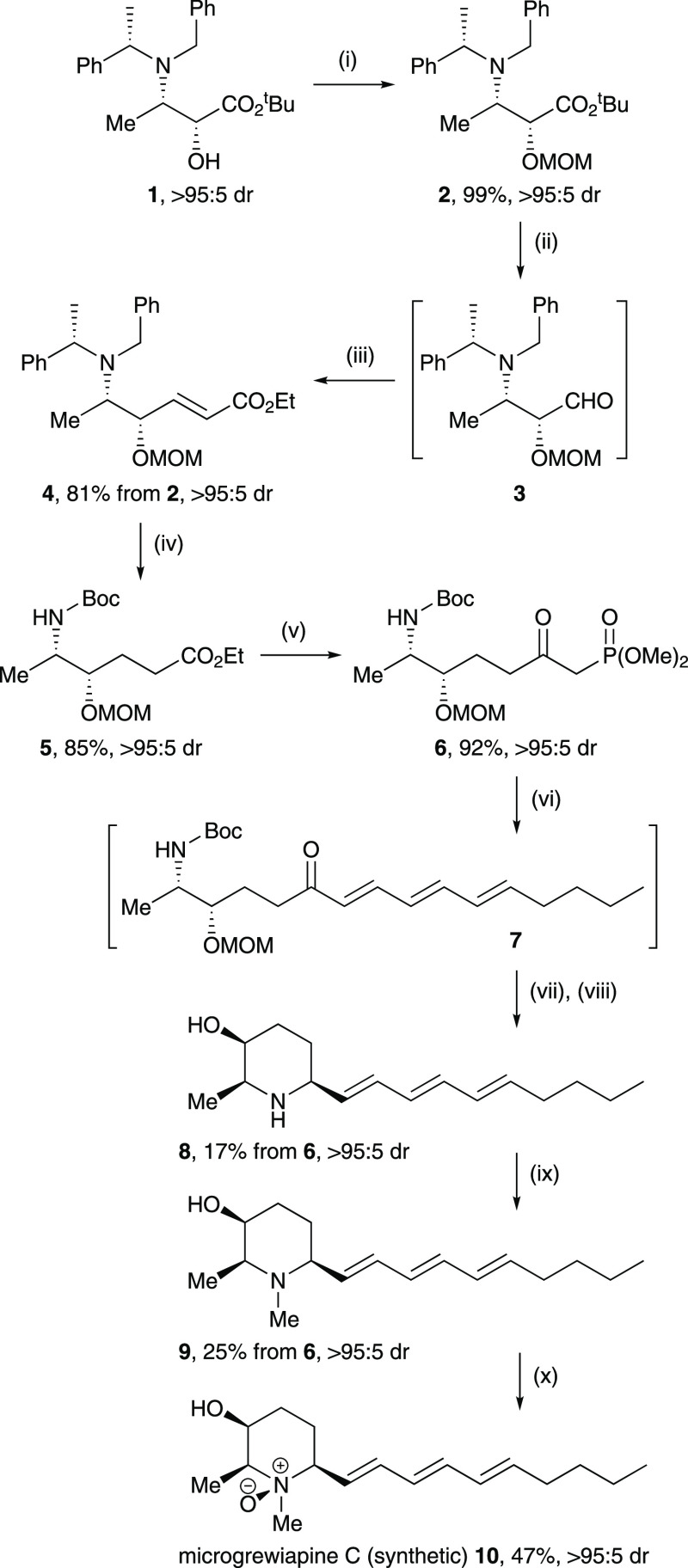
Preparation of Microgrewiapine C Reagents and conditions: (i) NaH, MOMCl, THF, 0 °C to rt, 12 h; (ii) DIBAL-H, CH_2_Cl_2_, −78 °C, 30 min; (iii) Ph_3_P=CHCO_2_Et, CH_2_Cl_2_, rt, 60 h; (iv) H_2_, Pd(OH)_2_/C, Boc_2_O, EtOAc, rt, 16 h; (v) BuLi, MeP(O)(OMe)_2_, THF, −78 to 0 °C, 90 min; (vi) NaH, (*E*,*E*)-2,4-nonadienal, THF, rt, 1 h, then 72 °C, 12 h; (vii) TFA, CH_2_Cl_2_, 0 °C, 10 min; (viii) NaBH_3_CN, concentrated aqueous HCl, CH_2_Cl_2_, EtOH, 0 °C, 30 min; (ix) formalin, NaBH_3_CN, MeCN, rt, 16 h; (x) *m*-CPBA, CHCl_3_, rt, 30 s.

The relative configurations within **8**–**10** were independently established by analysis of the relevant spectroscopic data. In this regard it is instructive at this point to recapitulate the characteristic data present in the corresponding 2,3-*trans*-3,6-*trans* diastereoisomers **11**–**13** that enabled confident assignment of their relative configurations.^9^ Our synthetic samples of microcosamine A (**11**), microgrewiapine A (**12**), and microgrewiapine B (**13**) displayed large ^1^H NMR ^3^*J* coupling constants between H-2 and H-3 (^3^*J*_2,3_ ≥ 8.8 Hz), consistent with an axial position for both within a chair conformation, and a reciprocal ^1^H–^1^H NMR NOE correlation between H-2 and H-6, consistent with an axial position for both; the 2,3-*trans*-3,6-*trans* relative configuration thus follows.^9^ Additional ^3^*J* coupling constants were resolved for **11**–**13** that provided further support for the assigned relative configurations, and a further series of reciprocal NOE interactions between the N*Me* group and both H-2 and H-6 within **13** suggested that the N*Me* group should be placed gauche (rather than anticoplanar) to both H-2 and H-6, i.e., in an equatorial position.^9^ Analysis of **8**–**10** using the same techniques revealed similar, reciprocal NOE correlations between H-2 and H-6, as well as between the N*Me* group and both H-2 and H-6. This strongly supported the assertion that **8**–**13** all share the common 2,6-*cis* relative configuration and that **10** and **13** share the same relative configuration at N-1, C-2, and C-6. An additional reciprocal NOE correlation between H-2 and H-3 within **8**–**10** suggested a gauche [H-2ax–H-3eq] relationship; it is noteworthy that such an interaction was not present for **11**–**13**, consistent with the assigned anticoplanar [H-2ax–H-3ax] relationship in these cases. In further support of this assignment, the ^3^*J* coupling constant between H-2 and H-3 was much smaller in **8**–**10** (^3^*J*_2,3_ ≈ 2 Hz) than in **11**–**13** (^3^*J*_2,3_ ≈ 9 Hz). All of these data can be interpreted on the basis of a chair conformation being adopted by the piperidine ring in solution, with H-2 and H-6 sited axial and H-3 sited equatorial; hence, the 2,3-*cis*-3,6-*cis* relative configuration of the three common stereogenic centers follows. As before, additional ^3^*J* coupling constants were resolved for **8**–**10**, which provided further support for the assigned relative configurations. Thus, from the 2,3-*cis*-3,6-*cis*-(1*RS*,2*SR*,3*SR*,6*SR*) relative configuration established for **10** and the established (2*R*,3*S*,α*S*) absolute configuration of **1**, it follows that the (1*R*,2*S*,3*S*,6*S*) absolute configuration can be unambiguously assigned to **10**. The stereochemical outcome of the intramolecular reductive amination of **7** (forming the diastereoisomeric product **8** with 2,6-*cis* relative configuration) is thus in accord with our previous results concerning the preparation of this family of alkaloids^8,9^ and those of other studies involving the intramolecular reductive amination of δ-amino ketones.^14^ It is of further note that *N*-oxidation of **9** gives *N*-oxide **10** as the only detectable diastereoisomeric product. This parallels our observation that *N*-oxidation of **12** gives *N*-oxide **13** as a single diastereoisomer. It may be tempting to explain both these observations as being the result of a completely diastereoselective *N*-oxidation pathway (particularly in the former case, where the *N*-oxidation of **9** may be directed by hydrogen bonding with the axial hydroxy substituent in the favored chair conformation),^15^ although the possibility of non-diastereoselective *N*-oxidation followed by decomposition (e.g., via a Cope elimination or [2,3]-Meisenheimer rearrangement) of the alternative *N*-oxide diastereoisomers (i.e., the N-1 epimers of **10** and **13**) to unknown products cannot be excluded by the data available.

**Figure 2:**
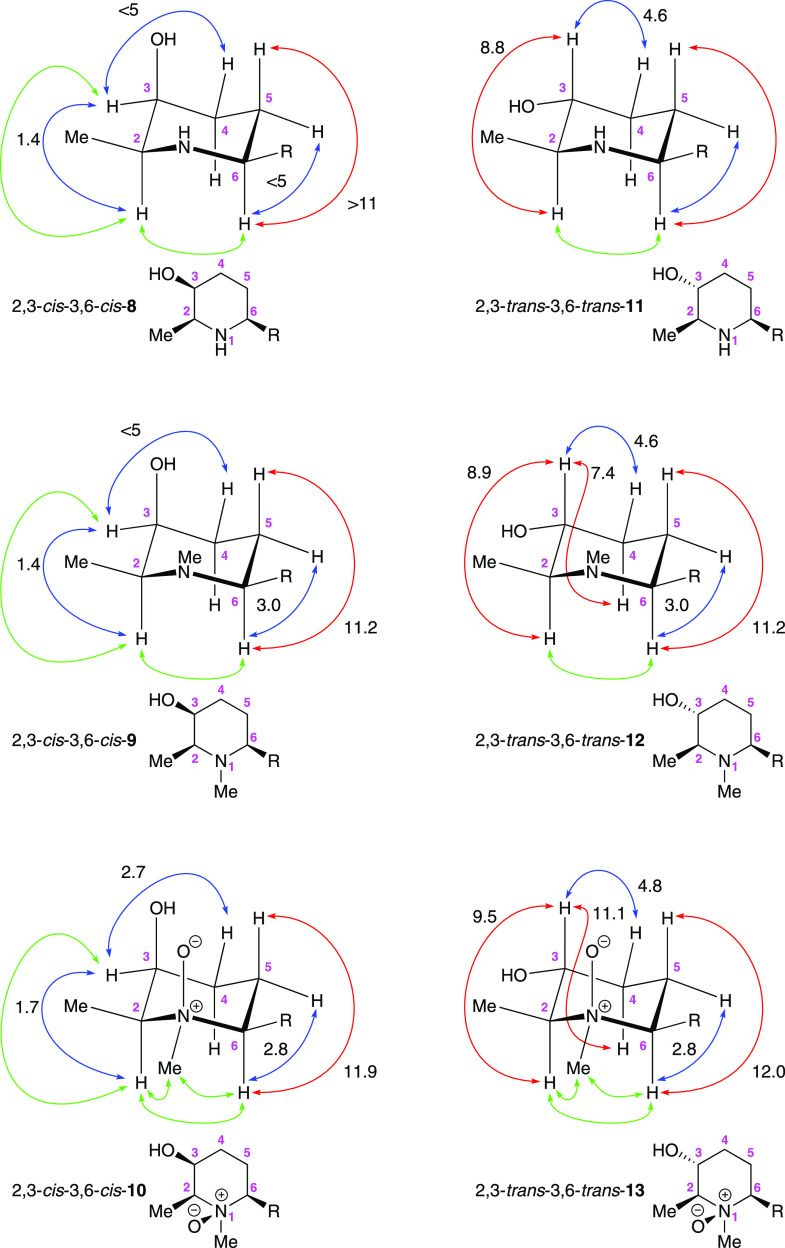
^1^H NMR ^3^*J* coupling constants (red arrows, φ ≈ 180°; blue arrows, φ ≈ 60°) and reciprocal NOE enhancements (green arrows) observed for 2,3-*cis*-3,6-*cis*-**8**–**10** compared to those in 2,3-*trans*-3,6-*trans*-**11**–**13**.

With the relative configuration of **10** securely established by analysis and interpretation of its spectroscopic data, comparison with the spectroscopic data reported for the natural product was next undertaken. We would like to express our gratitude to Professor Kinghorn and Dr. Still for supplying us with the raw data files for the various NMR spectra for microgrewiapine C, which thus allowed us to verify the data reported for the natural product, including the reference frequency of the spectra. Unfortunately, it transpired that there were some transcription errors in the originally presented data,^1^ and hence these data were first corrected (Table 1). Very good agreement was then noted when the corrected ^1^H and ^13^C NMR spectroscopic data for microgrewiapine C were compared with those for our sample **10** (Table 1). This analysis therefore unequivocally establishes that microgrewiapine C and **10** share the same relative configuration and that the natural product has the 2,3-*cis*-3,6-*cis*-(1*RS*,2*SR*,3*SR*,6*SR*) relative configuration, placing it into the same relative configuration series as microconine, as originally assigned.

**Table 1:** Corrected ^1^H and ^13^C NMR Data (CDCl_3_) for Microgrewiapine C (Kinghorn *et al.*, Ref (1)) and Data for the Synthetic Sample **10** (This Study)

	microgrewiapine C		**10**	
no.^a^	δ_C_^b^	δ_H_^b^^,^^c^	δ_C_^b^^,^^d^	δ_H_^b^^,^^c^
2	68.4	3.06 (app q, 5.8)	68.4 (0.0)	3.00 (qd, 6.5, 1.7)
3	70.9	3.83 (app br s)	71.0 (+0.1)	3.83 (app br s)
4ax	31.8	1.63 (m)	32.0 (+0.2)	1.65 (m)
4eq		2.01 (app br d, 13.2)		2.03 (app dq, 13.7, 2.7)
5-ax	23.7	2.67 (app qd, 13.4, 3.5)	23.8 (+0.1)	2.71 (app qd, 12.5, 3.9)
5-eq		1.55 (app br d, 15.4)		1.55 (app br d, 14.7)
6	79.2	3.51 (ddd, 12.2, 9.0, 2.4)	79.3 (+0.1)	3.46 (ddd, 11.9, 9.0, 2.8)
1′	127.6	5.99 (dd, 15.6, 9.0)	127.9 (+0.3)	6.03 (dd, 15.6, 9.0)
2′–5′	128.9	6.13 (m)^e^	129.0 (+0.1)	6.17 (m)^e^
	130.0		130.0 (0.0)	
	135.2		135.1 (−0.1)	
	135.8		135.6 (−0.2)	
6′	137.5	5.75 (dt, 14.5, 7.0)	137.4 (−0.1)	5.76 (dt, 14.7, 7.1)
7′	32.6	2.09 (app q, 6.6)	32.6 (0.0)	2.11 (app q, 6.8)
8′	31.4	1.32 (m)^e^	31.5 (+0.1)	1.32 (m)^e^
9′	22.3	1.32 (m)^e^	22.4 (+0.1)	1.32 (m)^e^
10′	14.0	0.88 (t, 7.0)	14.1 (+0.1)	0.89 (t, 7.2)
2-Me	13.4	1.65 (d, 6.5)	13.4 (0.0)	1.67 (d, 6.5)
NMe	53.8	2.86 (s)	54.0 (+0.2)	2.85 (s)

a Assignments are those made in this study.

b Reference frequencies employed are CHCl_3_, δ_H_ 7.26; CDCl_3_, δ_C_ 77.16 (ref (16)).

c Midpoints of all multiplets are quoted.

d Values of Δδ_C_ are given in parentheses [Δδ_C_ = δ_C_(synthetic) – δ_C_(natural)].

e Overlapping signals.

Attention was next directed toward the assignment of the absolute configuration of the alkaloid, as it was evidently nonracemic {[α]_D_^25^ +77.8 (*c* 0.1, MeOH)}.^1^ As we have previously noted that members of this family of piperidine-3-ol alkaloids display specific rotation values that are highly dependent (in both sign and magnitude) upon the concentration and temperature at which the value is measured (Table SI1, Supporting Information),^7,9^ the specific rotation value of **10** was determined under the same conditions (concentration and temperature, as well as solvent) as that for the natural product.^1^ The value so obtained for **10** {[α]_D_^25^ +77.1 (*c* 0.1, MeOH)} showed remarkable agreement of magnitude with that reported by Kinghorn et al. for microgrewiapine C,^1^ with the values displaying the same sign. These data suggest that **10** and the natural sample of the alkaloid microgrewiapine C have the same absolute configuration, which would indicate the (1*R*,2*S*,3*S*,6*S*) absolute configuration for the natural product, in fact, the absolute configuration originally suspected by Kinghorn et al. in their isolation study.^1^ However, this absolute configuration assignment places microgrewiapine C [(1*R*)-(2*S*,3*S*,6*S*)] into the opposite enantiomeric series to microconine [(2*R*,3*R*,6*R*)]. It therefore seems prudent to consider how these opposing absolute configuration assignments sit alongside biosynthetic considerations, given the origin of these two alkaloids from the same organism (different individuals from different regions of the globe, but members of the same biological taxon nonetheless). A similar issue has already arisen concerning the absolute configuration assignment of microgrewiapine A when considered alongside those of its congeners microcosamine A and microgrewiapine B.^1,9,10,17^ In this regard we advanced the hypothesis^10^ that the structural relationships of microcosamine A, microgrewiapine A, and microgrewiapine B are most readily rationalized (Ockham’s razor)^18^ if they are related as sequential intermediates on a biosynthetic pathway: initial biosynthesis of microcosamine A (from hitherto unknown precursors via an unknown route) followed by enzymatically mediated *N*-methylation would give microgrewiapine A, and then enzymatically mediated *N*-oxidation of microgrewiapine A would give microgrewiapine B. Thus, all three would share the same absolute configuration of the three common stereogenic centers around the piperidine ring.^10^ This assertion has very recently found credence in the results of the studies of Che, Ye, and Wang et al.,^5^ who reported the (re)isolation of microgrewiapine A (albeit from the leaves rather than the stem bark) of *M. paniculata*. They determined the structure and relative configuration of their sample of microgrewiapine A unambiguously by single-crystal X-ray diffraction analysis and assigned the (2*S*,3*R*,6*S*) absolute configuration to the alkaloid by comparison of its experimental ECD spectrum with the calculated ECD spectra [CAM-B3LYP/6-31+G(d)] of both enantiomers.^5^ The specific rotation value of their sample of microgrewiapine A {[α]_D_^25^ −22.8 (*c* 8.5, MeOH)}^5^ was, however, found to be opposite in sign to the value originally reported by Kinghorn et al. {[α]_D_^25^ +15.4 (*c* 0.1, MeOH)}^1^ for their sample of the alkaloid [note that although the concentrations of these samples are very different, their relative magnitudes are consistent with our previous observation of increasing magnitude of the specific rotation value of our synthetic sample of microgrewiapine A with increasing concentration of the sample {[α]_D_^25^ −16.0 (*c* 0.1, MeOH); [α]_D_^25^ −24.6 (*c* 0.5, MeOH); [α]_D_^25^ −26.2 (*c* 1.0, MeOH)}].^9^ As Kinghorn et al. had also assigned the (2*S*,3*R*,6*S*) absolute configuration to their sample of microgrewiapine A on the basis of the results of a Mosher’s ester analysis,^1^ it now seems almost certain that a typographical error of sign of the specific rotation value was made by Kinghorn et al. in the report of the original isolation study,^1^ as this is the only outlier from the significant weight of evidence now consistent with natural microgrewiapine A possessing the (2*S*,3*R*,6*S*) absolute configuration; hence it seems that microcosamine A, microgrewiapine A, and microgrewiapine B are indeed “three homochiral alkaloids”.^9^

In the present case, given the similarities in the structures of microconine and microgrewiapine C, it is possible to conjecture another such simple biosynthetic relationship, this time involving **9** as a common biosynthetic precursor to both alkaloids: enzyme-mediated *N*-oxidation of **9** would give **10**, and enzyme-mediated *O*-methylation of **9** would give **14** (Figure 3).^19^ As there has been no in-depth study into the biosynthetic origin of these or other piperidine-3-ol alkaloids closely related in structure, it is of course also possible that the enantiomer **9′** serves as the common biosynthetic precursor to **10′** and **14′**. Either way, the configurational assignments for microgrewiapine C and microconine made solely on the basis of comparison of reported specific rotation values, thus equating them to **10** and **14′**, respectively, seem very unlikely when the potential of such a common biosynthetic origin is considered. This again identifies a need for reisolation of these alkaloids from their natural source to confirm their specific rotation values, in particular the sign of both of these values, either of which may have been erroneously reported (as is seemingly the case with the sign of the specific rotation value reported by Kinghorn et al. for microgrewiapine A).^1,5^ It is also of note that the sign of the specific rotation value of our synthetic sample of **14** has been observed to change with the concentration of the sample {[α]_D_^22^ −29.0 (*c* 1.0, CHCl_3_); [α]_D_^22^ −20.9 (*c* 0.1, CHCl_3_); [α]_D_^22^ −8.6 (*c* 0.06, CHCl_3_); [α]_D_^22^ +7.2 (*c* 0.04, CHCl_3_)}, and the concentration at which the value for the natural product was obtained was not given in the original report {[α]_D_^22^ +29.2 (CHCl_3_)}. Given the various issues that have been noted during our studies into these alkaloids (inconsistent reports of specific rotations and variation of the sign and magnitude of the values of the specific rotations of these alkaloids), it is promulgated that ideally both specific rotation values and ECD spectra should be reported for any new compounds such that similar inconsistencies do not occur in the future; the reisolation of these alkaloids should thus also allow for a more detailed investigation of their absolute configuration by ECD analysis, which should resolve these discrepancies.

**Figure 3:**
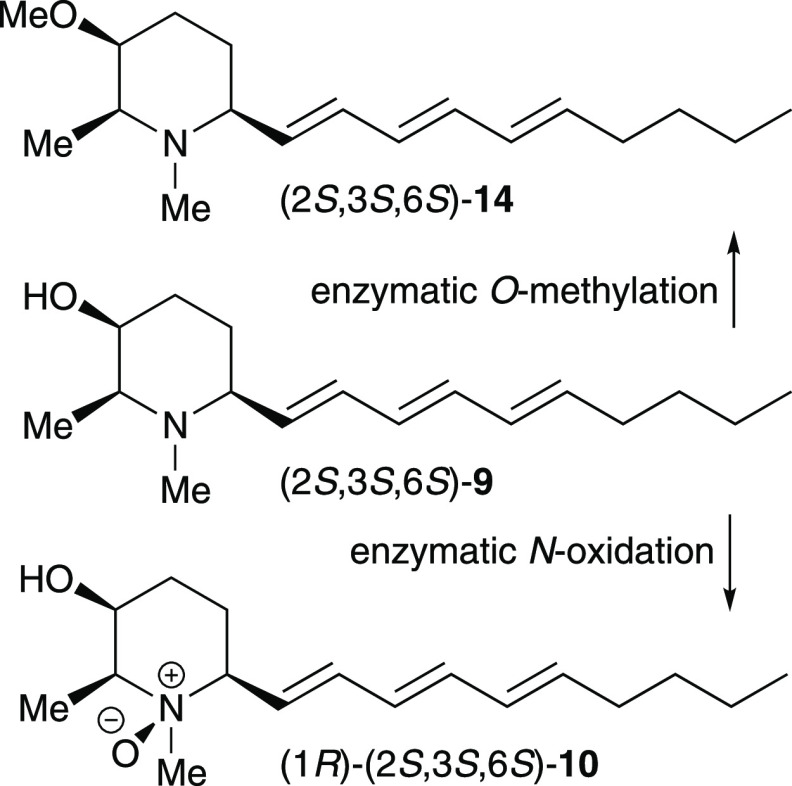
Plausible biosynthetic origin consistent with the co-occurrence of microgrewiapine C and microconine within the same organism.

In conclusion, the first asymmetric synthesis of microgrewiapine C has been developed, and the ^1^H and ^13^C NMR data for the natural sample have been corrected. Comparison of the data for the natural product and the synthetic sample unambiguously establish the 2,3-*cis*-3,6-*cis* relative configuration of the alkaloid. Although comparison of specific rotation values suggests that the alkaloid possesses the (1*R*,2*S*,3*S*,6*S*) absolute configuration, a lack of knowledge of the biosynthetic origins of these compounds casts some doubt on this assignment when it is considered alongside the assigned absolute configurations of other very structurally similar alkaloids that have been isolated from the same organism. It is therefore advanced that there is a need for reisolation of these alkaloids to verify the signs of the specific rotations and for a more detailed investigation into the biosynthetic origins of these compounds to be undertaken before firm conclusions are drawn into their absolute configurations. It is hoped that the results of our studies concerning the configuration assignments (both relative and absolute) of this family of alkaloids will prove insightful and instructive for other future studies involving configuration assignments of newly discovered members of this family—and indeed other disparate families—of fascinating natural products.

## Experimental Section

### General Experimental Procedures

Specific rotations are reported in 10^–1^ deg cm^2^ g^–1^ and concentrations in g/100 mL. IR spectra were recorded using an ATR module. Selected characteristic peaks are reported in cm^–1^. NMR spectra were recorded in CDCl_3_. Reference frequencies employed were as follows: CHCl_3_, δ_H_ = 7.26; CDCl_3_, δ_C_ = 77.16.^16^^1^H–^1^H COSY and ^1^H–^13^C HSQC analyses were used to establish atom connectivity. Accurate mass measurements were run on a MicroTOF instrument internally calibrated with polyalanine. CH_2_Cl_2_ was dried according to the procedure outlined by Grubbs and co-workers.^20^

### *tert*-Butyl (2*R*,3*S*,α*S*)-2-Methoxymethyloxy-3-[*N*-benzyl-*N*-(α-methylbenzyl)amino]butanoate **2**

NaH (60% dispersion in mineral oil, 116 mg, 1.60 mmol) was stirred in THF (10.1 mL) for 30 min. The resultant suspension was then cooled to 0 °C, a solution of **1** (890 mg, 2.41 mmol)^7,11,12^ in THF (2.0 mL) was added, and the resultant solution was stirred at 0 °C for 30 min. MOMCl (0.22 mL, 2.9 mmol) was then added, and the resultant solution was allowed to warm to rt over 12 h. H_2_O (12 mL) was then added, and the aqueous layer was extracted with Et_2_O (3 × 10 mL). The combined organics were washed with brine (20 mL), then dried (MgSO_4_) and concentrated in vacuo to give **2** as a yellow oil (981 mg, 99%, >95:5 dr); [α]_D_^25^ −9.7 (*c* 1.0, CHCl_3_); ν_max_ 2974, 2931, 1737; δ_H_ (400 MHz, CDCl_3_) 1.14 (3H, d, *J* 7.0, H_3_-4), 1.31 (3H, d, *J* 6.9, Me-α), 1.43 (9H, s, CMe_3_), 3.25–3.39 (1H, m, H-3) overlapping 3.26 (3H, s, OMe), 3.78 (1H, d, *J* 14.7, NCH_B_), 3.83 (1H, d, *J* 5.9, H-2), 3.94 (1H, d, *J* 14.7, NCH_A_), 4.15 (1H, q, *J* 6.9, H-α), 4.41 (1H, d, *J* 6.8, OCH_B_), 4.53 (1H, d, *J* 6.8, OCH_A_), 7.16–7.49 (10H, m, Ph); δ_C_ (100 MHz, CDCl_3_) 14.1 (C-4), 17.6 (Me-α), 28.1 (C*Me*_3_), 50.6 (NCH_2_), 55.6 (C-3), 56.2 (OMe), 59.2 (C-α), 81.5 (*C*Me_3_), 96.6 (OCH_2_), 126.6, 126.7 (*p*-Ph), 128.0, 128.1, 128.6 (*o*,*m*-Ph), 142.3, 145.0 (*i*-Ph), 171.2 (C-1); HRESIMS *m*/*z* 436.2464 [M + Na]^+^ (calcd for C_25_H_35_NNaO_4_^+^, 436.2458).

### Ethyl (4*S*,5*S*,α*S*,*E*)-4-Methoxymethyloxy-5-[*N*-benzyl-*N*-(α-methylbenzyl)amino]hex-2-enoate, **4**

Step 1. DIBAL-H (1.0 M in PhMe, 45 mL, 45 mmol) was added to a stirred solution of **2** (6.27 g, 15.2 mmol) in CH_2_Cl_2_ (150 mL) at −78 °C, and the resultant solution was stirred at −78 °C for 30 min. MeOH (90 mL) was then added, and the resultant solution was allowed to warm to rt before saturated aqueous Rochelle salt (90 mL) and CH_2_Cl_2_ (90 mL) were added sequentially. The resultant mixture was stirred at rt for 16 h. The aqueous layer was then extracted with CH_2_Cl_2_ (3 × 90 mL), and the combined organics were washed with brine (50 mL), then dried (MgSO_4_) and concentrated in vacuo to give **3** as a yellow oil (4.73 g, > 95:5 dr); δ_H_ (400 MHz, CDCl_3_) 1.34 (3H, d, *J* 6.9, H_3_-4), 1.38 (3H, d, *J* 7.0, Me-α), 3.36 (3H, s, OMe), 3.51 (1H, qd, *J* 6.9, 4.0, H-3), 3.65 (1H, d, *J* 13.6, NCH_B_), 3.81 (1H, dd, *J* 4.0, 0.7, H-2), 4.00 (1H, q, *J* 7.0, H-α), 4.22 (1H, d, *J* 13.6, NCH_A_), 4.59 (1H, d, *J* 6.9, OCH_B_), 4.64 (1H, d, *J* 6.9, OCH_A_), 7.19–7.40 (10H, m, Ph), 8.86 (1H, d, *J* 13.6, H-1).

Step 2. Ph_3_P=CHCO_2_Et (7.41 g, 21.3 mmol) was added to a stirred solution of **3** (4.84 g, 14.2 mmol) in CH_2_Cl_2_ (37 mL) at rt, and the resultant solution was stirred at rt for 60 h before being concentrated in vacuo. Purification via flash column chromatography on silica gel (eluent 30–40 °C petrol/Et_2_O, 10:1, increased to 30–40 °C petrol/Et_2_O, 7:3) gave **4** as a yellow oil (5.20 g, 81% from **2**, >95:5 dr [(*E*):(*Z*) ratio]); [α]_D_^25^ −65.4 (*c* 1.0, CHCl_3_); ν_max_ 2990, 2886, 1723; δ_H_ (400 MHz, CDCl_3_) 1.12 (3H, d, *J* 7.0, H_3_-6), 1.22 (3H, t, *J* 7.1, OCH_2_C*H*_3_), 1.28 (3H, d, *J* 6.7, Me-α), 3.01 (1H, qd, *J* 7.0, 4.5, H-5), 3.11 (3H, s, OMe), 3.68 (1H, d, *J* 14.3, NCH_B_), 3.85 (1H, ddd, *J* 6.1, 4.5, 1.4, H-4), 3.93 (1H, d, *J* 14.3, NCH_A_), 3.96 (1H, q, *J* 6.7, H-α), 4.11 (2H, q, *J* 7.1, OC*H*_2_CH_3_), 4.27 (1H, d, *J* 6.6, OCH_B_), 4.35 (1H, d, *J* 6.6, OCH_A_), 5.82 (1H, dd, *J* 15.8, 1.3, H-2), 6.79 (1H, dd, *J* 15.8, 6.1, H-3), 7.09–7.44 (10H, m, *Ph*); δ_C_ (100 MHz, CDCl_3_) 13.5, 13.7 (C-6, Me-α), 14.4 (OCH_2_*C*H_3_), 51.5 (NCH_2_), 54.8 (C-5), 55.7 (OMe), 56.7 (C-α), 60.4 (O*C*H_2_CH_3_), 80.2 (C-4), 94.9 (OCH_2_), 122.6 (C-2), 126.7, 126.9 (*p*-Ph), 128.0, 128.2, 128.4, 128.7 (*o*,*m*-Ph), 141.5, 144.3 (*i*-Ph), 146.7 (C-3), 166.2 (C-1); HRESIMS *m*/*z* 434.2237 [M + Na]^+^ (calcd for C_25_H_33_NNaO_4_^+^, 434.2302).

### Ethyl (4*S*,5*S*)-4-Methoxymethyloxy-5-(*N*-*tert*-butoxycarbonylamino)hexanoate, **5**

Pd(OH)_2_/C (50% w/w of **4**, 2.25 g) was added to a stirred solution of **4** (4.50 g, 10.9 mmol) and Boc_2_O (7.96 g, 36.5 mmol) in degassed EtOAc (42 mL), and the resultant black suspension was stirred at rt under a hydrogen atmosphere (5 atm) for 16 h. After depressurization, the reaction mixture was filtered through a plug of Celite (eluent CH_2_Cl_2_) and concentrated in vacuo. Purification via flash column chromatography on silica gel (eluent 30–40 °C petrol/Et_2_O, 3:1, increased to 30–40 °C petrol/Et_2_O, 2:1) gave **5** as a yellow oil (2.96 g, 85%, >95:5 dr); [α]_D_^25^ −15.8 (*c* 1.0, CHCl_3_); ν_max_ (film) 3402, 2967, 1738, 1711; δ_H_ (500 MHz, CDCl_3_) 1.15 (3H, d, *J* 6.8, H_3_-6), 1.23 (3H, t, *J* 7.1, OCH_2_C*H*_3_), 1.42 (9H, s, CMe_3_), 1.73–1.87 (2H, m, H_2_-3), 2.29–2.46 (2H, m, H_2_-2), 3.38 (3H, s, OMe), 3.47 (1H, td, *J* 6.6, 2.9, H-4), 3.71–3.82 (1H, m, H-5), 4.11 (2H, q, *J* 7.1, OC*H*_2_CH_3_), 4.62 (1H, d, *J* 6.9, OCH_B_), 4.66 (1H, d, *J* 6.9, OCH_A_), 4.71 (1H, br d, *J* 6.0, NH); δ_C_ (125 MHz, CDCl_3_) 14.3 (OCH_2_*C*H_3_), 18.1 (C-6), 26.6 (C-3), 28.5 (C*Me*_3_), 30.4 (C-2), 48.3 (C-5), 56.1 (OMe), 60.5 (O*C*H_2_CH_3_), 79.2 (*C*Me_3_), 80.0 (C-4), 96.8 (OCH_2_), 155.6 (*C*O_2_^t^Bu), 173.4 (C-1); HRESIMS *m*/*z* 342.1888 [M + Na]^+^ (calcd for C_15_H_29_NNaO_6_^+^, 342.1887).

### (5*S*,6*S*)-1-(Dimethoxyphosphoryl)-5-methoxymethyloxy-6-(*N*-*tert*-butoxycarbonylamino)heptan-2-one, **6**

BuLi (2.5 M in hexanes, 3.31 mL, 8.28 mmol) was added dropwise to a stirred solution of dimethyl methyl phosphonate (0.89 mL, 8.28 mmol) in THF (24 mL) at −78 °C. The resultant solution was stirred at −78 °C for 30 min; then a solution of **5** (1.06 g, 3.31 mmol) in THF (4.9 mL) was added dropwise. The resultant solution was stirred at −78 °C for 30 min, before being allowed to warm to 0 °C over 1 h. Saturated aqueous NH_4_Cl (1 mL) was then added, and the aqueous layer was extracted with EtOAc (3 × 30 mL). The combined organics were dried (MgSO_4_) and concentrated in vacuo. Purification via flash column chromatography on silica gel (eluent 30–40 °C petrol/Et_2_O, 1:1, increased to 30–40 °C petrol/acetone, 7:3) gave **6** as a colorless oil (1.21 g, 92%, >95:5 dr); [α]_D_^25^ −21.0 (*c* 1.0, CHCl_3_); ν_max_ 3309, 2973, 1715, 1265; δ_H_ (400 MHz, CDCl_3_) 1.15 (3H, d, *J* 6.8, H_3_-7), 1.43 (9H, s, CMe_3_), 1.70–1.86 (2H, m, H_2_-4), 2.71 (2H, app q, *J* 7.4, H_2_-3), 3.08 (1H, d, *J* 22.5, H-1b), 3.11 (1H, d, *J* 22.5, H-1a), 3.39 (3H, s, OMe), 3.45 (1H, td, *J* 6.6, 2.9, H-5), 3.70–3.81 (6H, m, H-6, POMe_2_), 4.61 (1H, d, *J* 6.8, OCH_B_), 4.65–4.72 (2H, m, OCH_A_, NH); δ_C_ (100 MHz, CDCl_3_) 18.1 (C-7), 24.9 (C-4), 28.5 (C*Me*_3_), 40.2 (C-3)), 41.4 (d, *J* 128.4, C-1), 48.1 (C-6), 53.1 (d, *J* 6.6, POMe_2_), 56.0 (OMe), 79.3 (*C*Me_3_), 79.8 (C-5), 96.7 (OCH_2_), 155.7 (*C*O_2_^t^Bu), 201.5 (d, *J* 6.4, C-2); HRESIMS *m*/*z* 420.1759 [M + Na]^+^ (calcd for C_16_H_32_NNaO_8_P^+^, 420.1758).

### (2*S*,3*S*,6*S*,1′*E*,3′*E*,5′*E*)-2-Methyl-6-(deca-1′,3′,5′-trienyl)piperidin-3-ol, **8**

Step 1. NaH (60% dispersion in mineral oil, 14 mg, 0.36 mmol) was stirred in THF (0.66 mL) at rt for 5 min. The resultant suspension was then cooled to 0 °C, a solution of **6** (139 mg, 0.349 mmol) in THF (1.36 mL) was added, and the resultant solution was stirred at rt for 15 min. A solution of (*E*,*E*)-2,4-nonadienal (71 mg, 0.52 mmol) in THF (1.11 mL) was then added, and the resultant solution was stirred at rt for 1 h. The resultant solution was then heated to 72 °C and stirred at 72 °C for 12 h. The resultant solution was allowed to cool to rt, then poured into a mixture of saturated aqueous brine (7 mL) and H_2_O (7 mL). The aqueous layer was extracted with EtOAc (3 × 10 mL); then the combined organics were dried (MgSO_4_) and concentrated in vacuo. The residue was dissolved in DMF (3.0 mL), and saturated aqueous NaHSO_3_ (6.0 mL) was added. The resultant suspension was stirred at rt for 10 min, then diluted with H_2_O (6 mL). The resultant solution was extracted with Et_2_O (2 × 6 mL), and the combined organics were washed with H_2_O (2 × 4 mL, then 1 mL), then dried (Na_2_SO_4_) and concentrated in vacuo.

Step 2. The residue from the previous step was dissolved in CH_2_Cl_2_ (11.3 mL), and the resultant solution was cooled to 0 °C. TFA (6.0 mL) was added dropwise, and the resultant solution was stirred at 0 °C for 10 min. The resultant solution was then concentrated in vacuo.

Step 3. The residue from the previous step was dissolved in CH_2_Cl_2_ (15 mL); the resultant solution and concentrated aqueous HCl (2.4 mL) were then simultaneously added to a stirred suspension of NaBH_3_CN (302 mg, 4.79 mmol) in EtOH (30 mL) at 0 °C. The resultant suspension was stirred at 0 °C for 15 min; then further portions of NaBH_3_CN (145 mg, 2.30 mmol) and concentrated aqueous HCl (1.5 mL) were added sequentially. The resultant suspension was stirred at 0 °C for 15 min; then H_2_O (10 mL) was added. The resultant mixture was poured into a mixture of saturated aqueous NaHCO_3_ (150 mL) and H_2_O (150 mL). The aqueous layer was extracted with CH_2_Cl_2_ (3 × 100 mL); then the combined organics were dried (Na_2_SO_4_) and concentrated in vacuo. The residue was then dissolved in 1.25 M methanolic HCl (3.2 mL) at rt, and the resultant solution was stirred at rt for 12 h. The resultant solution was then poured into 1 M aqueous KOH (6 mL), the aqueous layer was extracted with CHCl_3_/^i^PrOH (v/v, 3:1, 4 × 6 mL), and then the combined organics were dried (Na_2_SO_4_) and concentrated in vacuo. Purification via flash column chromatography on silica gel (eluent CHCl_3_/(7 M NH_3_ in MeOH solution), 99:1) gave a yellow oil (*R*_*f*_ = 0.42, eluent CHCl_3_/(7 M NH_3_ in MeOH solution), 9:1). This was dissolved in 6 M aqueous HCl (2.0 mL), and the resultant solution was washed with Et_2_O (3 × 2 mL), then basified by the addition of 2 M aqueous NaOH until pH > 14 was achieved. The resultant solution was extracted with Et_2_O (3 × 3 mL); then the combined organics were dried (Na_2_SO_4_) and concentrated in vacuo to give **8** as a white solid (15 mg, 17% from **6**, > 95:5 dr); [α]_D_^25^ +6.2 (*c* 0.1, CHCl_3_); ν_max_ 3352, 3349, 2956, 2926; δ_H_ (500 MHz, CDCl_3_) 0.89 (3H, t, *J* 7.2, H_3_-10′), 1.11 (3H, d, *J* 6.5, Me-2), 1.27–1.40 (4H, m, H_2_-8′, H_2_-9′), 1.45–1.57 (2H, m, H-4b, H-5b), 1.90–1.95 (1H, m, H-4a), 2.00–2.06 (1H, m, H-5a), 2.09 (2H, app q, *J* 7.0, H_2_-7′), 2.81 (1H, qd, *J* 6.5, 1.4, H-2), 3.17–3.22 (1H, m, H-6), 3.53–3.56 (1H, m, H-3), 5.61 (1H, dd, *J* 15.3, 7.2, H-1′), 5.70 (1H, dt, *J* 14.6, 7.0, H-6′), 6.01–6.21 (4H, m, H-2′, H-3′, H-4′, H-5′); δ_C_ (125 MHz, CDCl_3_) 14.1 (C-10′), 18.8 (Me-2), 22.4 (C-9′), 26.6 (C-5), 31.6 (C-8′), 32.0 (C-4), 32.6 (C-7′), 55.7 (C-2), 59.6 (C-6), 67.6 (C-3), 130.0, 130.3, 130.4, 133.1 (C-2′, C-3′, C-4′, C-5′), 135.6, 135.8 (C-1′, C-6′); HRESIMS *m*/*z* 250.2167 [M + H]^+^ (calcd for C_16_H_28_NO^+^, 250.2165).

### (2*S*,3*S*,6*S*,1′*E*,3′*E*,5′*E*)-*N*-Methyl-2-methyl-6-(deca-1′,3′,5′-trienyl)piperidin-3-ol, **9**

Step 1. NaH (60% dispersion in mineral oil, 11.1 mg, 0.278 mmol) was stirred in THF (0.52 mL) at rt for 5 min. The resultant suspension was then cooled to 0 °C, a solution of **6** (110 mg, 0.277 mmol) in THF (1.08 mL) was added, and the resultant solution was stirred at rt for 15 min. A solution of (*E*,*E*)-2,4-nonadienal (56 mg, 0.41 mmol) in THF (0.88 mL) was then added, and the resultant suspension was stirred at rt for 1 h. The resultant solution was then heated to 72 °C and stirred at 72 °C for 12 h. The resultant solution was allowed to cool to rt, then poured into a mixture of saturated aqueous brine (5 mL) and H_2_O (5 mL). The aqueous layer was extracted with EtOAc (3 × 10 mL); then the combined organics were dried (MgSO_4_) and concentrated in vacuo. The residue was dissolved in DMF (2 mL), and saturated aqueous NaHSO_3_ (5 mL) was added. The resultant suspension was stirred at rt for 10 min, then diluted with H_2_O (5 mL). The resultant solution was extracted with Et_2_O (2 × 5 mL), and the combined organics were washed with H_2_O (2 × 3 mL, then 1 mL), then dried (Na_2_SO_4_) and concentrated in vacuo.

Step 2. The residue from the previous step was dissolved in CH_2_Cl_2_ (9.0 mL), and the resultant solution was cooled to 0 °C. TFA (4.8 mL) was added dropwise, and the resultant solution was stirred at 0 °C for 10 min. The resultant solution was then concentrated in vacuo.

Step 3. The residue from the previous step was dissolved in CH_2_Cl_2_ (11.5 mL); the resultant solution and concentrated aqueous HCl (1.9 mL) were then simultaneously added to a stirred suspension of NaBH_3_CN (240 mg, 3.81 mmol) in EtOH (24 mL) at 0 °C. The resultant suspension was stirred at 0 °C for 15 min; then further portions of NaBH_3_CN (115 mg, 1.83 mmol) and concentrated aqueous HCl (1.2 mL) were added sequentially. The resultant suspension was stirred at 0 °C for 15 min; then H_2_O (10 mL) was added. The resultant mixture was poured into a mixture of saturated aqueous NaHCO_3_ (120 mL) and H_2_O (120 mL). The aqueous layer was extracted with CH_2_Cl_2_ (3 × 100 mL); then the combined organics were dried (Na_2_SO_4_) and concentrated in vacuo. The residue was then dissolved in 1.25 M methanolic HCl (2.6 mL) at rt, and the resultant solution was stirred at rt for 12 h. The resultant solution was then poured into 1 M aqueous KOH (5 mL), and the aqueous layer was extracted with CHCl_3_/^i^PrOH (v/v, 3:1, 4 × 5 mL); then the combined organics were dried (Na_2_SO_4_) and concentrated in vacuo.

Step 4. The residue from the previous step was dissolved in MeCN (13 mL), and 35% aqueous HCHO (0.54 mL, 4.83 mmol) was added. The resultant solution was cooled to 0 °C, and NaBH_3_CN (51 mg, 0.81 mmol) was added. The resultant solution was allowed to warm to rt and then stirred at rt for 16 h. The resultant solution was poured into 1 M aqueous NaOH (15 mL) and then extracted with CHCl_3_ (3 × 15 mL). The combined organics were washed with brine (20 mL), then dried (Na_2_SO_4_) and concentrated in vacuo. Purification via flash column chromatography on silica gel (eluent CHCl_3_/(7 M NH_3_ in MeOH solution), 98:2) gave a yellow oil (*R*_*f*_ = 0.36, eluent CHCl_3_/(7 M NH_3_ in MeOH solution), 9:1). This was dissolved in 6 M aqueous HCl (2.0 mL), and the resultant solution was washed with Et_2_O (3 × 2 mL), then basified by the addition of 2 M aqueous NaOH until pH > 14 was achieved. The resultant solution was washed with Et_2_O (3 × 3 mL); then the combined organics were dried (Na_2_SO_4_) and concentrated in vacuo to give **9** as a yellow solid (18 mg, 25% from **6**, >95:5 dr); [α]_D_^25^ −15.2 (*c* 1.0, CHCl_3_); ν_max_ 3650, 3024, 2925; δ_H_ (500 MHz, CDCl_3_) 0.89 (3H, t, *J* 7.2, H_3_-10′), 1.19 (3H, d, *J* 6.5, Me-2), 1.26–1.39 (4H, m, H_2_-8′, H_2_-9′), 1.42–1.46 (1H, m, H-5b), 1.53 (1H, dddd, *J* 13.4, 4.7, 2.5, H-4ax), 1.69–1.78 (1H, m, H-5a), 1.82–1.88 (1H, m, H-4eq), 2.07–2.12 (2H, m, H_2_-7′), 2.12–2.16 (1H, m, H-2), 2.14 (3H, s, NMe), 2.49 (1H, ddd, *J* 11.7, 8.8, 3.2, H-6), 3.52–3.61 (1H, m, H-3), 5.54 (1H, dd, *J* 14.2, 8.8, H-1′), 5.70 (1H, dt, *J* 14.5, 7.1, H-6′), 6.01–6.17 (4H, m, H-2′, H-3′, H-4′, H-5′); δ_C_ (125 MHz, CDCl_3_) 14.1 (C-10′), 18.4 (Me-2), 22.4 (C-9′), 28.1 (C-5), 31.5, 31.6 (C-4, C-8′), 32.6 (C-7′), 40.0 (NMe), 62.5 (C-2), 68.4 (C-6), 70.4 (C-3), 130.1, 130.3, 131.2, 132.7 (C-2′, C-3′, C-4′, C-5′), 135.7 (C-6′), 136.8 (C-1′); HRESIMS *m*/*z* 264.2322 [M + H]^+^ (calcd for C_17_H_29_NO^+^, 264.2322).

### (1*R*,2*S*,3*S*,6*S*,1′*E*,3′*E*,5′*E*)-*N*-Methyl-2-Methyl-6-(deca-1′,3′,5′-trienyl)piperidin-3-ol-*N*-oxide, **10**

*m*-CPBA (33% by wt, 17 mg, 0.08 mmol) was added to a stirred solution of **9** (20 mg, 0.08 mmol) in CHCl_3_ (0.8 mL)^21^ at rt, and the resultant solution was stirred at rt for 30 s. Et_3_N (0.07 mL, 0.46 mmol) was then added, and the resultant solution was concentrated in vacuo. The residue was dissolved in CH_2_Cl_2_ (5 mL), and the resultant solution was washed with saturated aqueous NaHCO_3_ (5 mL), then concentrated in vacuo. Purification via flash column chromatography on activated basic alumina (eluent CHCl_3_, then CHCl_3_/MeOH, 3:1) gave a white solid (*R*_*f*_ = 0.59, eluent CHCl_3_/MeOH, 3:1). Further purification via flash column chromatography on silica gel (eluent CHCl_3_/MeOH/Et_3_N, 14:1:0.1) gave **10** as a white solid (10 mg, 47%, >95:5 dr); [α]_D_^25^ +77.1 (*c* 1.0, MeOH); ν_max_ 3377, 2956, 2926, 1672; δ_H_ (500 MHz, CDCl_3_) 0.89 (3H, t, *J* 7.2, H_3_-10′), 1.24–1.39 (4H, m, H_2_-8′, H_2_-9′), 1.55 (1H, app br d, 14.7, H-5eq), 1.60–1.69 (1H, m, H-4ax) overlapping 1.67 (3H, d, *J* 6.5, Me-2), 2.03 (1H, app dq, *J* 13.7, 2.7, H-4eq), 2.11 (2H, app q, *J* 6.8, H_2_-7′), 2.71 (1H, app qd, *J* 12.5, 3.9, H-5ax), 2.85 (3H, s, NMe), 3.00 (1H, qd, *J* 6.5, 1.7, H-2), 3.46 (1H, ddd, *J* 11.9, 9.0, 2.8, H-6), 3.83 (1H, app br s, H-3), 5.76 (1H, dt, *J* 14.7, 7.1, H-6′), 6.03 (1H, dd, *J* 15.6, 9.0, H-1′), 6.09–6.25 (4H, m, H-2′, H-3′, H-4′, H-5′); δ_C_ (125 MHz, CDCl_3_) 13.4 (Me-2), 14.1 (C-10′), 22.4 (C-9′), 23.8 (C-5), 31.5 (C*-*8′), 32.0 (C-4), 32.6 (C-7′), 54.0 (NMe), 68.4 (C-2), 71.0 (C-3), 79.3 (C-6), 127.9 (C-1′), 129.0, 130.0, 135.1, 135.6 (C-2′, C-3′, C-4′, C-5′), 137.4 (C-6′); HRESIMS *m*/*z* 280.2270 [M + H]^+^ (calcd for C_17_H_30_NO_2_^+^, 280.2271).

## Supporting Information Available

Copies of ^1^H and ^13^C NMR spectra; Table SI1 (specific rotation values for synthetic samples of the alkaloids and related precursors) (PDF)
