# Supplementary material for: Microgrewiapine C: Asymmetric Synthesis, Spectroscopic Data, and Configuration Assignment
Source: J Nat Prod. 2022 Jun 30;85(7):1872–9. doi: 10.1021/acs.jnatprod.2c00183 (PMC9315977; doi:10.1021/acs.jnatprod.2c00183)
Supplement: Supplementary file 1 — np2c00183_si_001.pdf [file np2c00183_si_001.pdf]

Supporting Information for

Microgrewiapine C: Asymmetric Synthesis, Spectroscopic Data and Configuration Assignment

Stephen G. Davies,* Ai M. Fletcher, Paul M. Roberts,
Cameron E. Taylor, and James E. Thomson

Department of Chemistry, Chemistry Research Laboratory,
University of Oxford, Mansfield Road, Oxford OX1 3TA, U.K.

steve.davies@chem.ox.ac.uk

Table of Contents

Copies of ^{1}H and ^{13}C NMR Spectra S2–S15

Table SI1 S16

***tert*-Butyl (2*R*,3*S*,α*S*)-2-methoxymethyloxy-3-[*N*-benzyl-*N*-(α-methylbenzyl)amino]butanoate 2 (400 MHz, ^{1}H, CDCl3)**

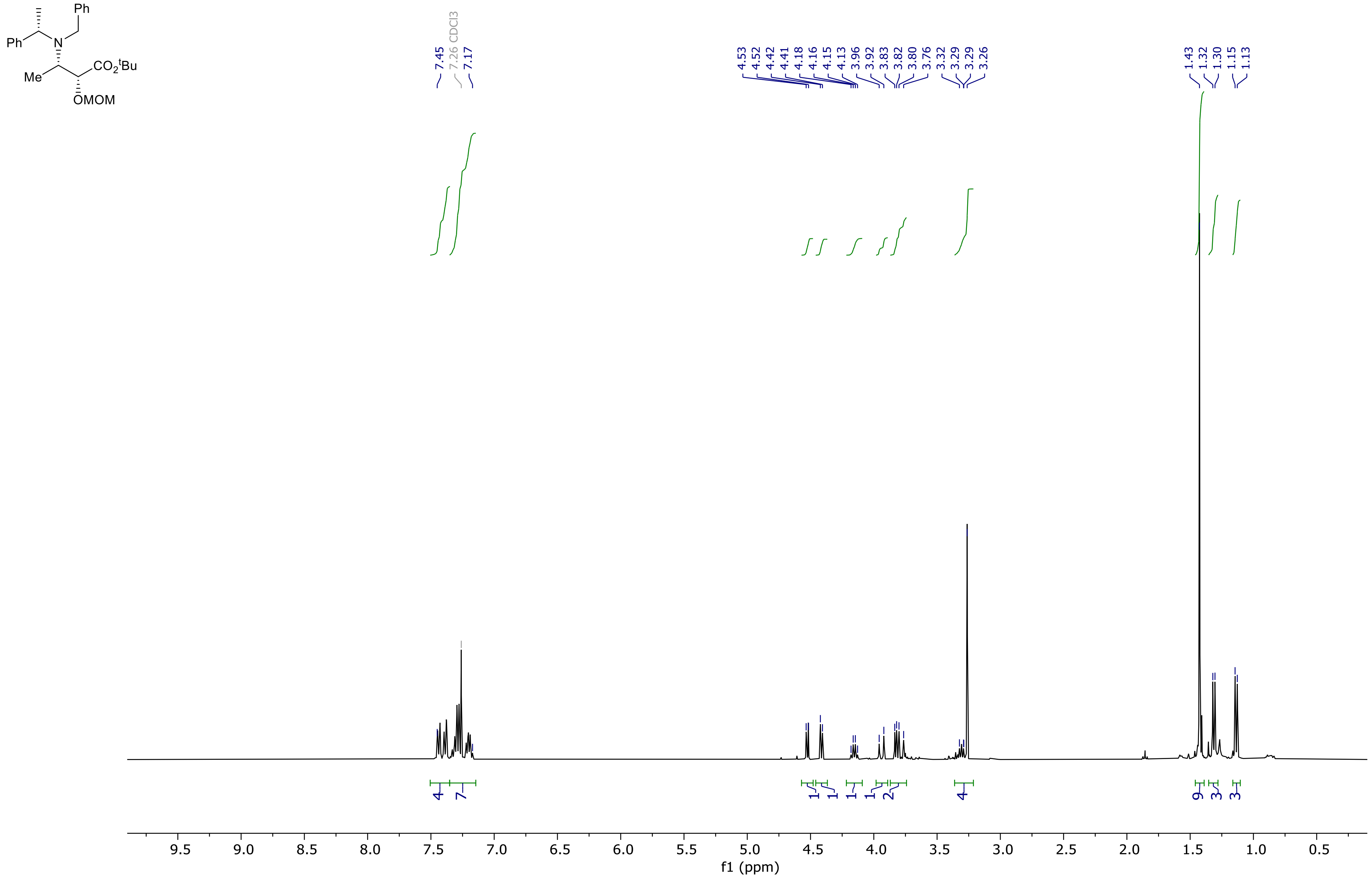

tert-Butyl (2*R*,3*S*,α*S*)-2-methoxymethyloxy-3-[*N*-benzyl-*N*-(α-methylbenzyl)amino]butanoate 2 (100 MHz, ^{13}C, CDCl$_3$)

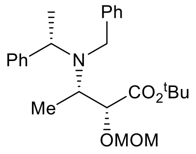

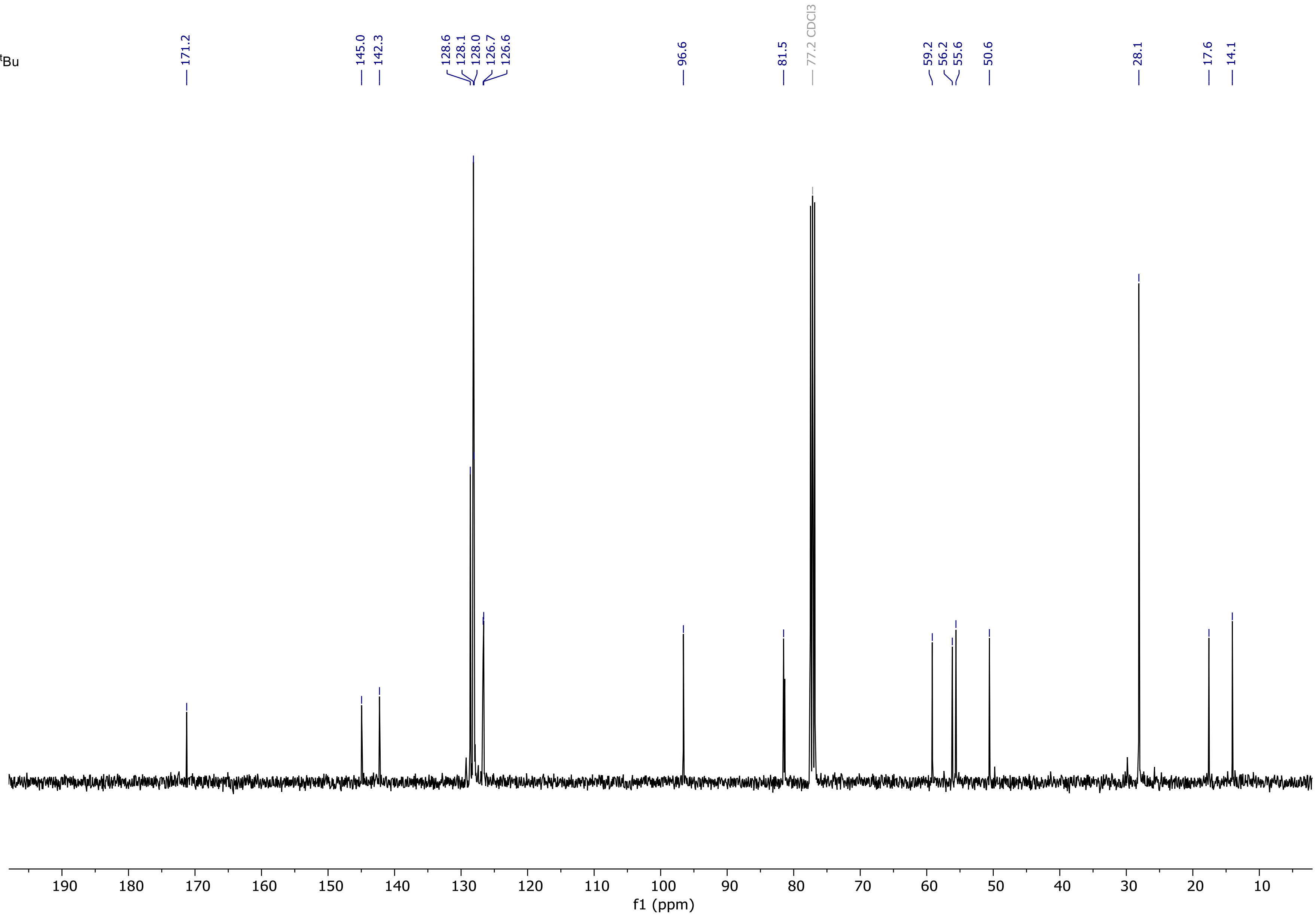

Ethyl (4*S*,5*S*,α*S*,*E*)-4-methoxymethyloxy-5-[*N*-benzyl-*N*-(α-methylbenzyl)amino]hex-2-enoate 4 (400 MHz, ^{1}H, $CDCl_3$)

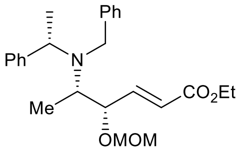

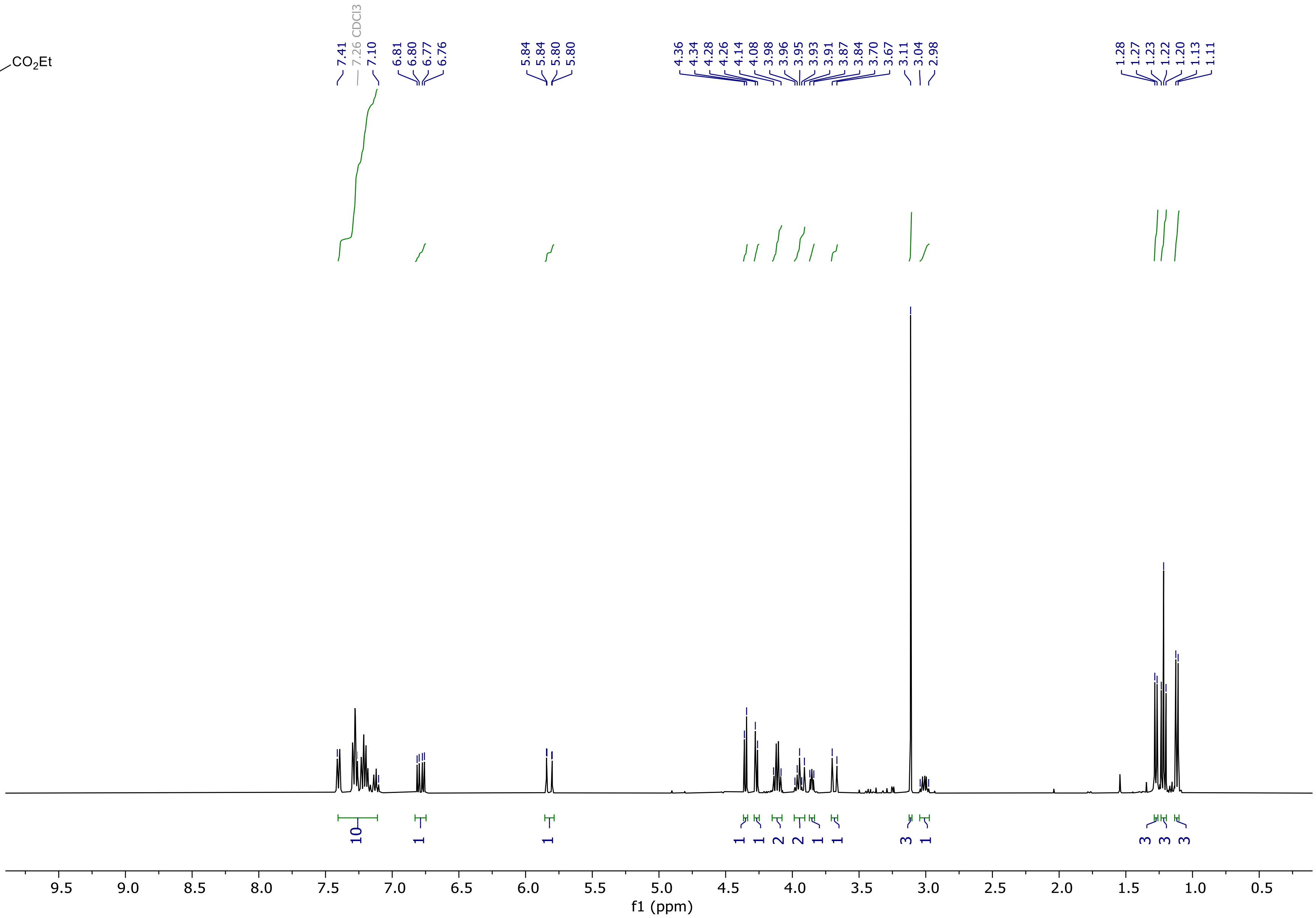

Ethyl (4*S*,5*S*,α*S*,*E*)-4-methoxymethyloxy-5-[*N*-benzyl-*N*-(α-methylbenzyl)amino]hex-2-enoate 4 (100 MHz, ^{13}C, $CDCl_3$)

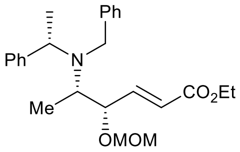

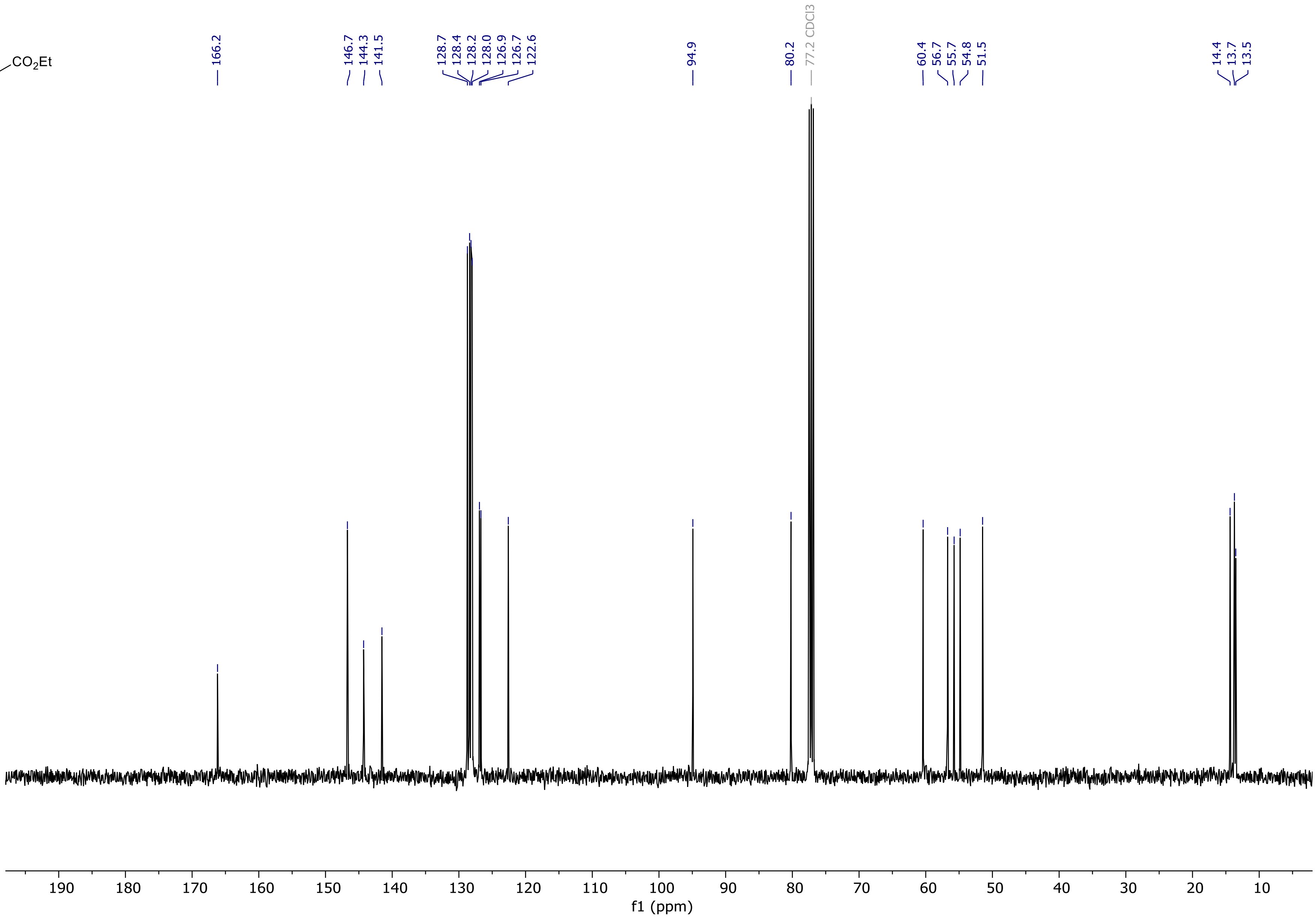

Ethyl (4*S*,5*S*)-4-methoxymethyloxy-5-(*N*-*tert*-butoxycarbonylamino)hexanoate 5 (500 MHz, ^{1}H, $CDCl_3$)

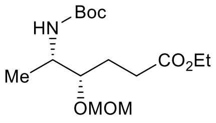

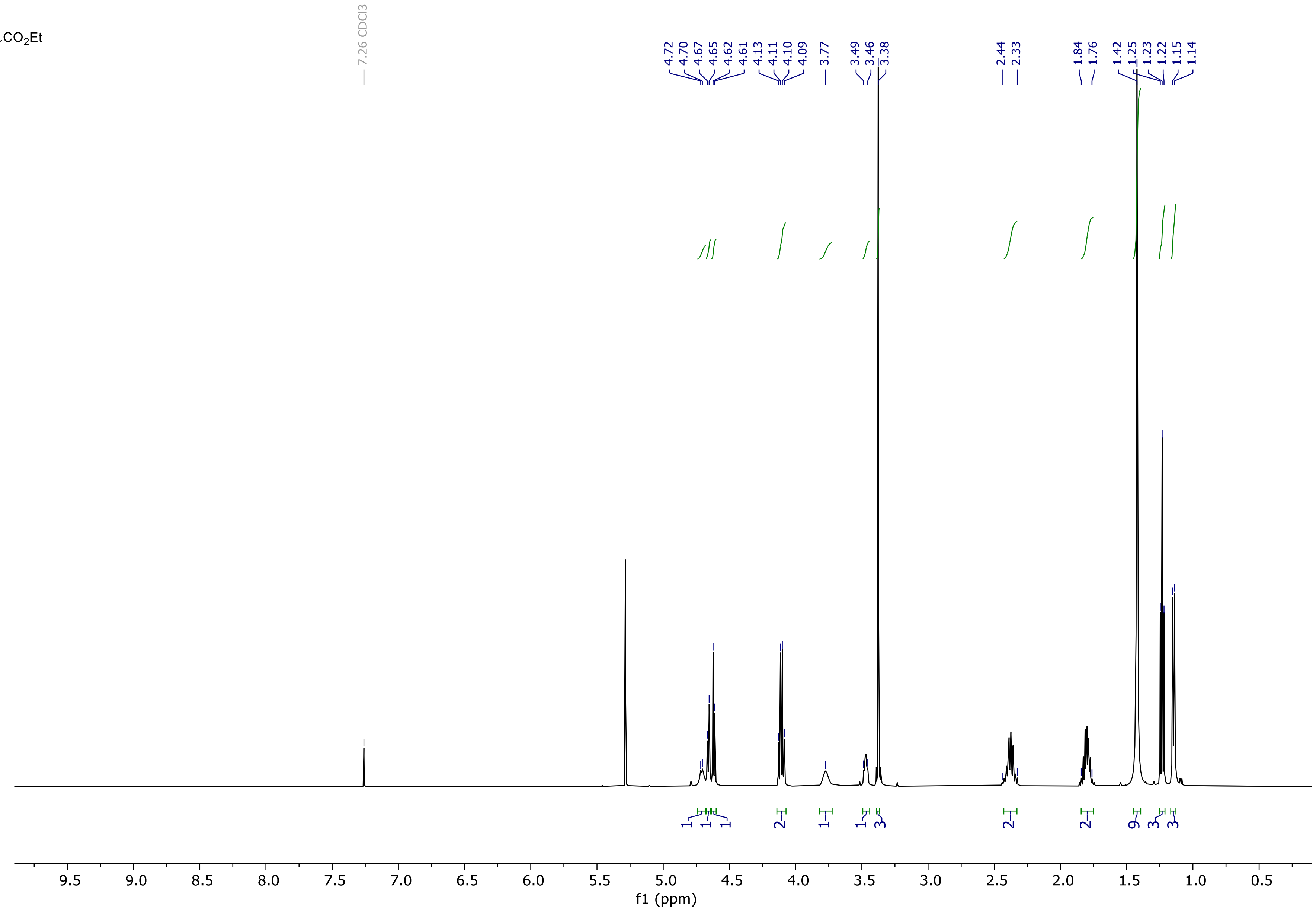

Ethyl (4*R*,5*S*)-4-methoxymethyloxy-5-(*N-tert*-butoxycarbonylamino)hexanoate 5 (125 MHz, ^{13}C, $CDCl_3$)

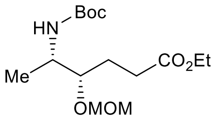

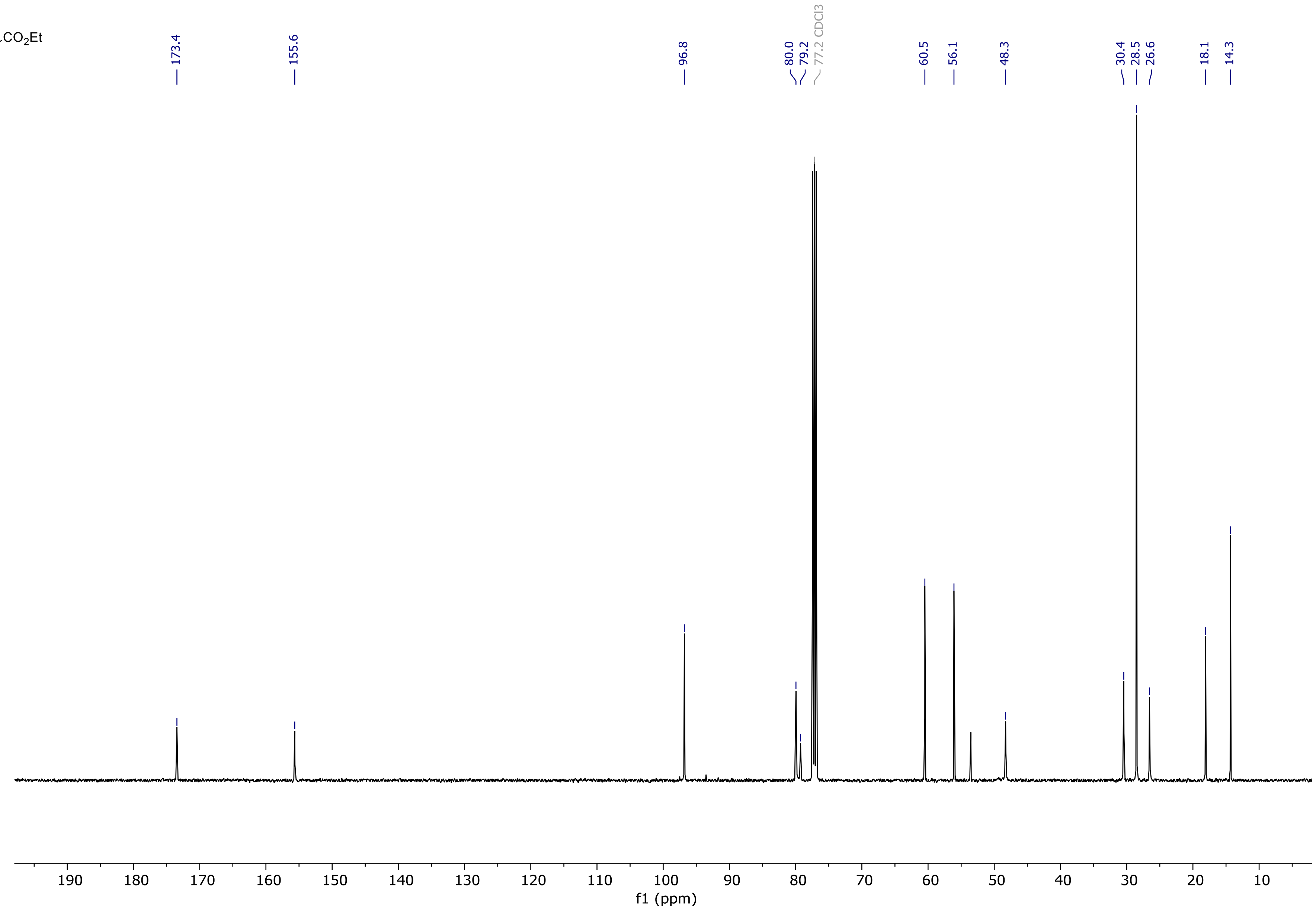

(5*S*,6*S*)-1-(Dimethoxyphosphoryl)-5-methoxymethyloxy-6-(*N*-*tert*-butoxycarbonylamino)heptan-2-one 6 (400 MHz, ^{1}H, CDCl$_3$)

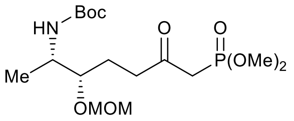

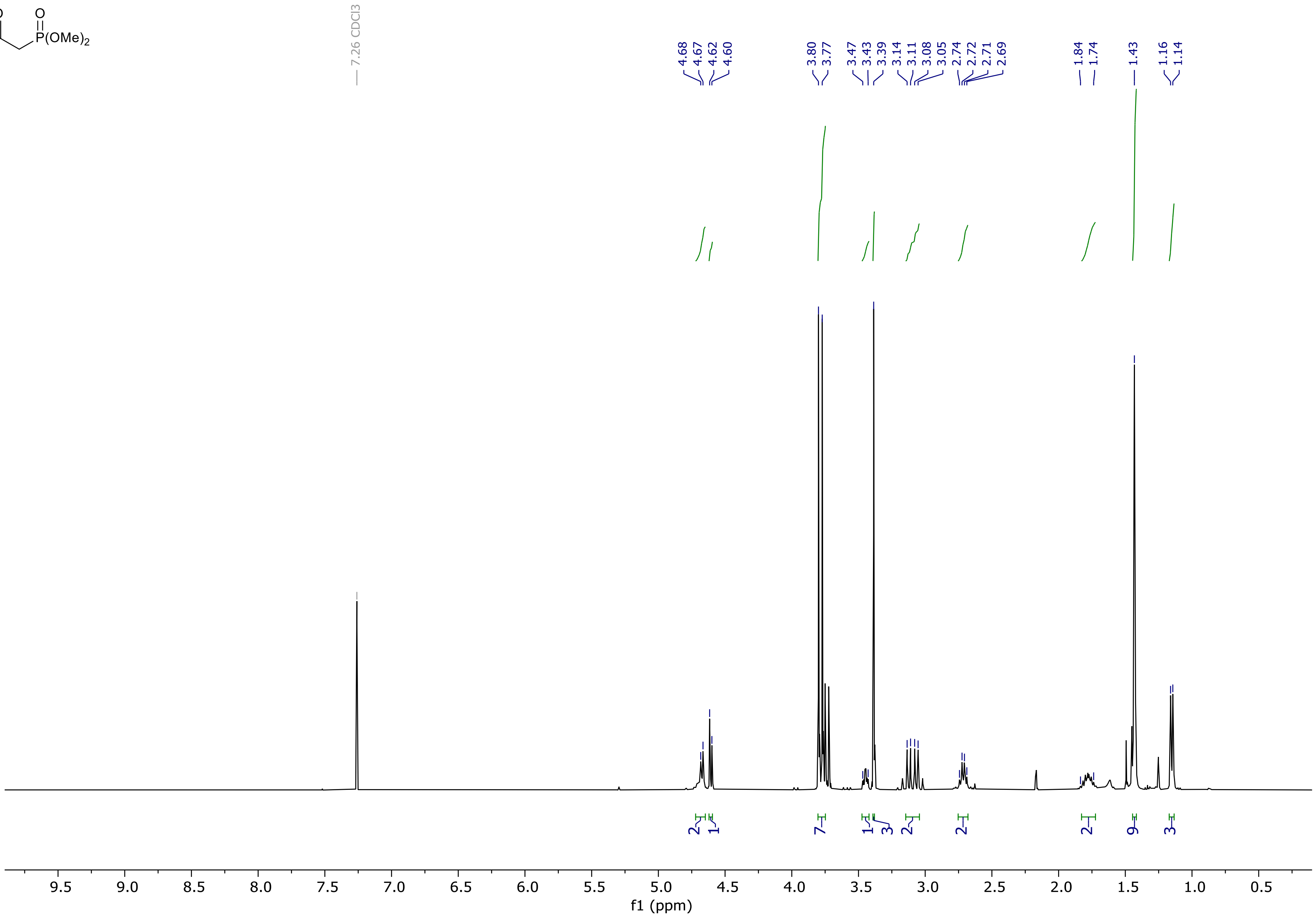

(5*S*,6*S*)-1-(Dimethoxyphosphoryl)-5-methoxymethyloxy-6-(*N*-*tert*-butoxycarbonylamino)heptan-2-one 6 (100 MHz, ^{13}C, $CDCl_3$)

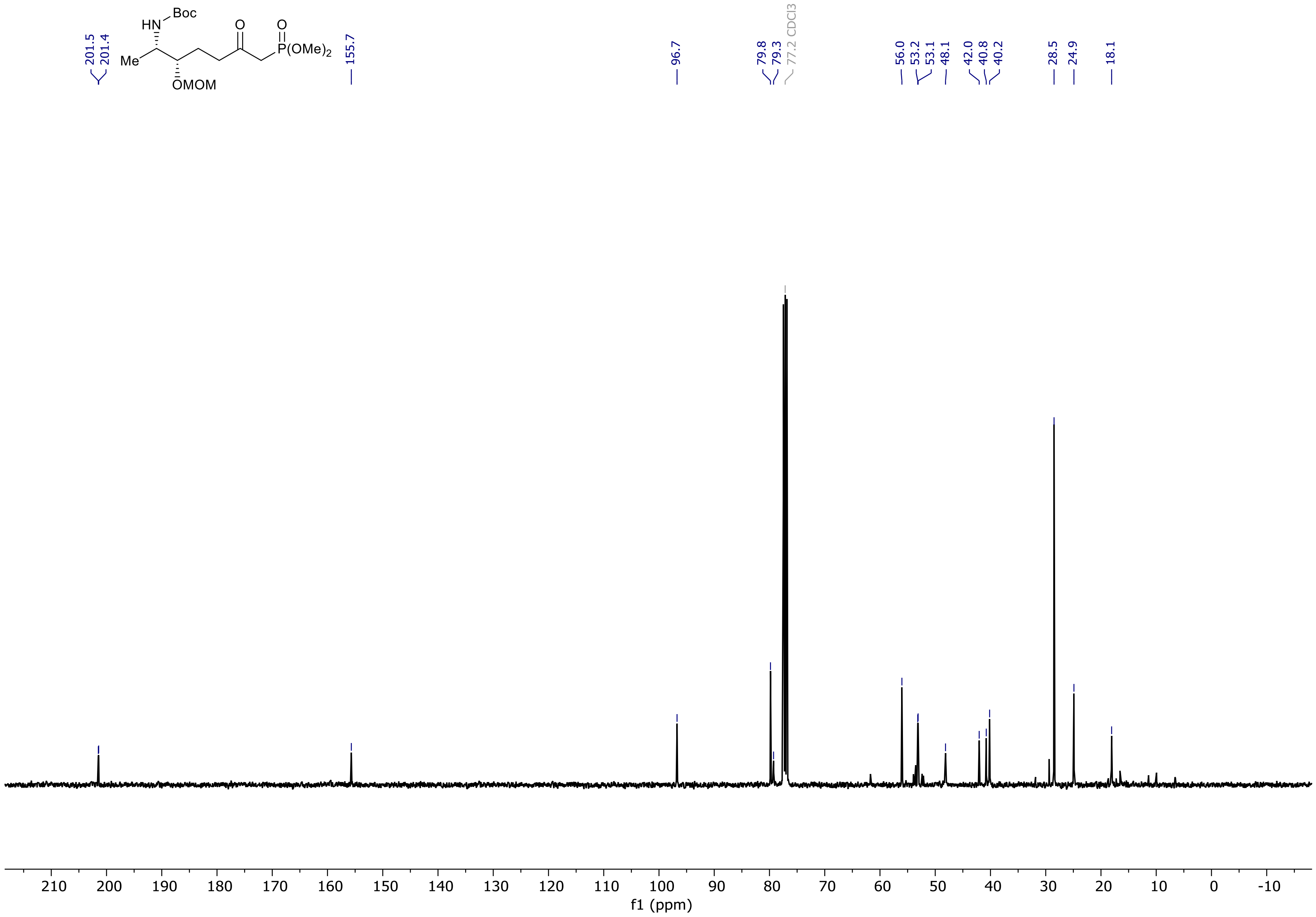

(2*S*,3*S*,6*S*,1′*E*,3′*E*,5′*E*)-2-Methyl-6-(deca-1′,3′,5′-trienyl)piperidin-3-ol 8 (500 MHz, ^{1}H, $CDCl_3$)

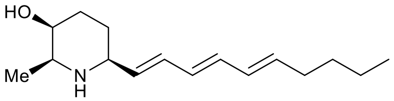

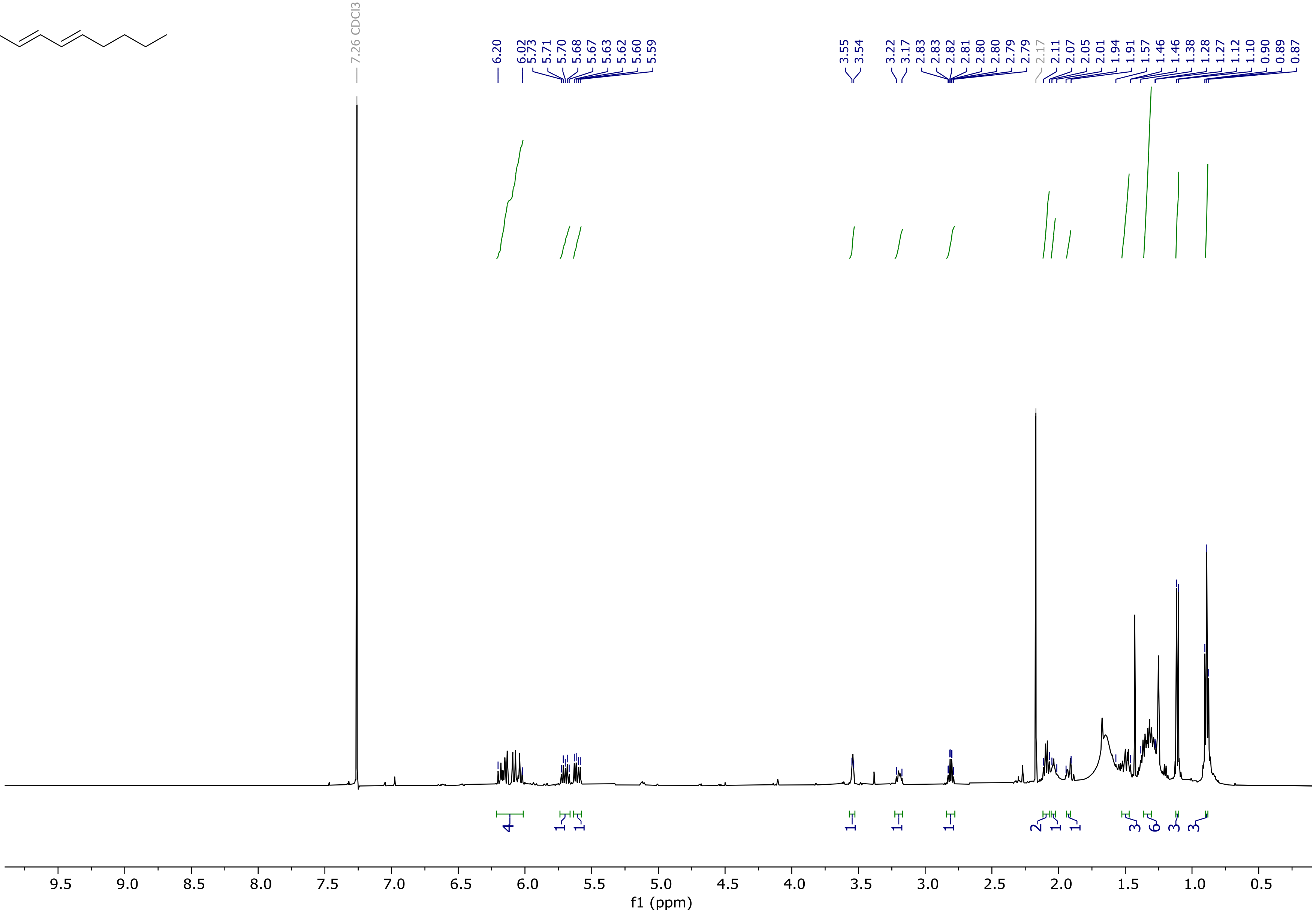

(2*S*,3*S*,6*S*,1′*E*,3′*E*,5′*E*)-2-Methyl-6-(deca-1′,3′,5′-trienyl)piperidin-3-ol 8 (125 MHz, ^{13}C, $CDCl_3$)

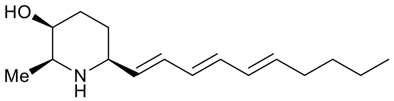

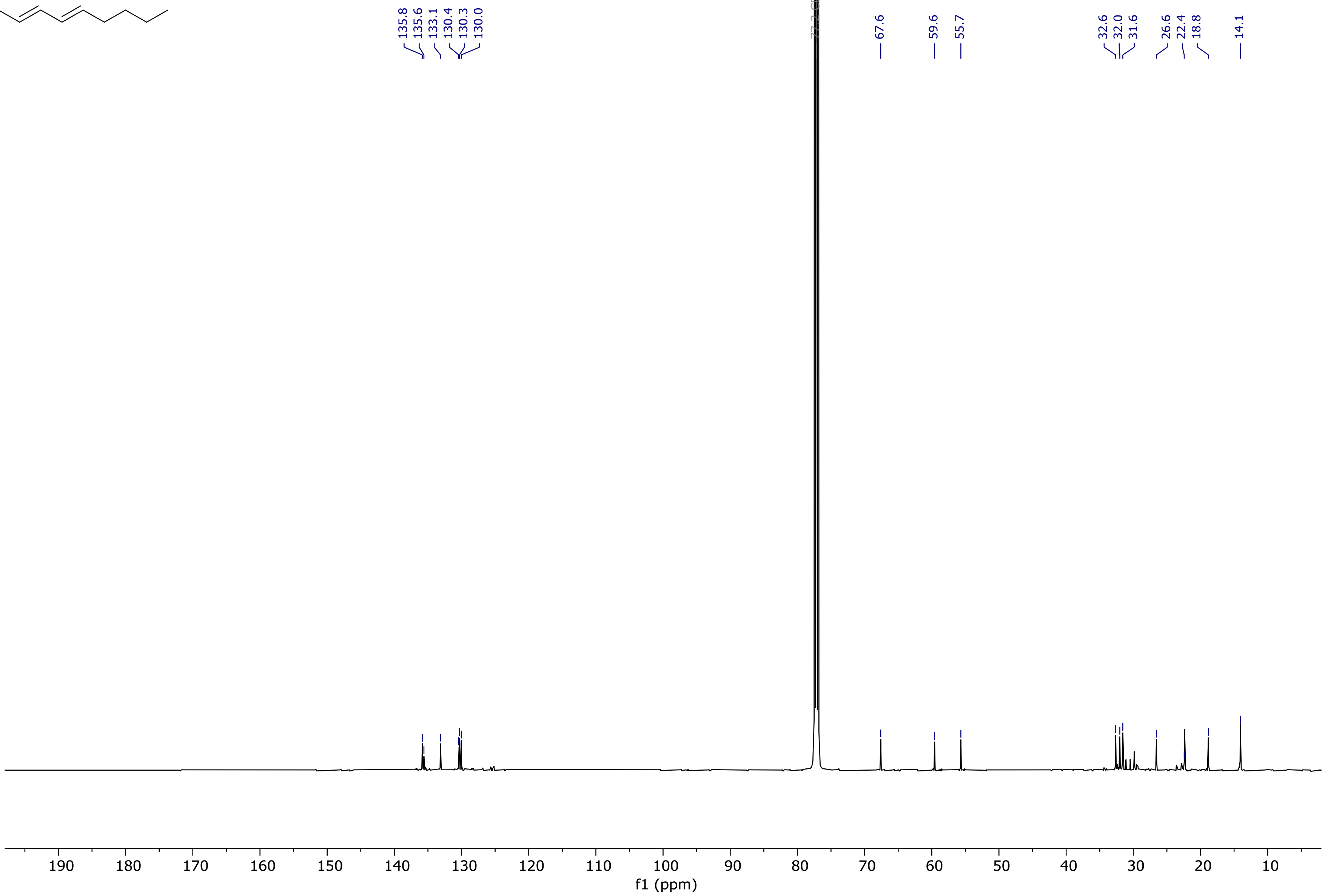

(2*S*,3*S*,6*S*,1′*E*,3′*E*,5′*E*)-*N*-Methyl-2-Methyl-6-(deca-1′,3′,5′-trienyl)piperidin-3-ol 9 (500 MHz, ^{1}H, $CDCl_3$)

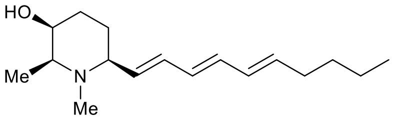

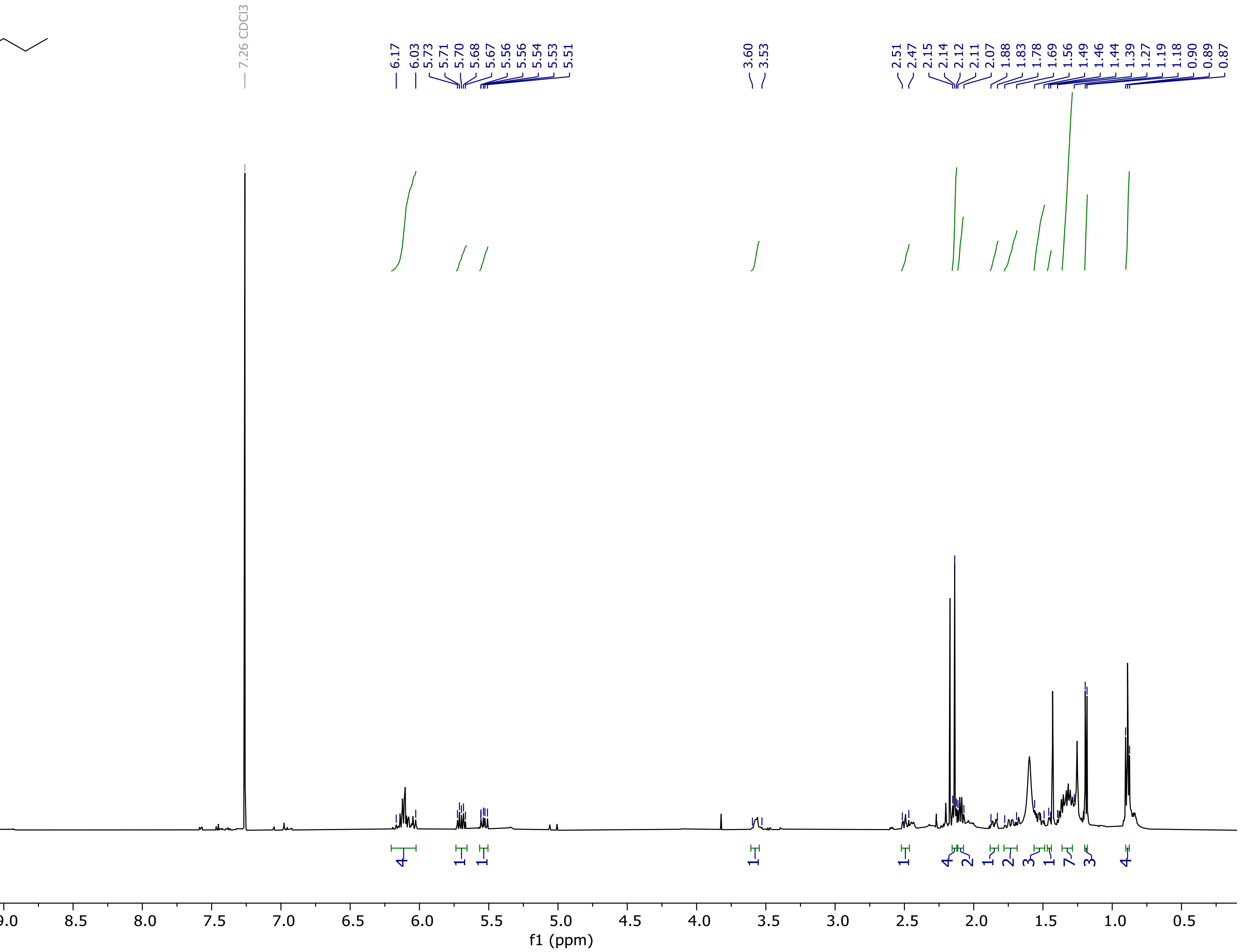

(2*S*,3*S*,6*S*,1′*E*,3′*E*,5′*E*)-*N*-Methyl-2-Methyl-6-(deca-1′,3′,5′-trienyl)piperidin-3-ol 9 (125 MHz, ^{13}C, $CDCl_3$)

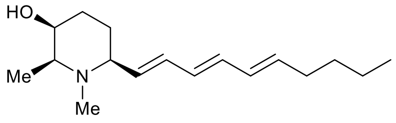

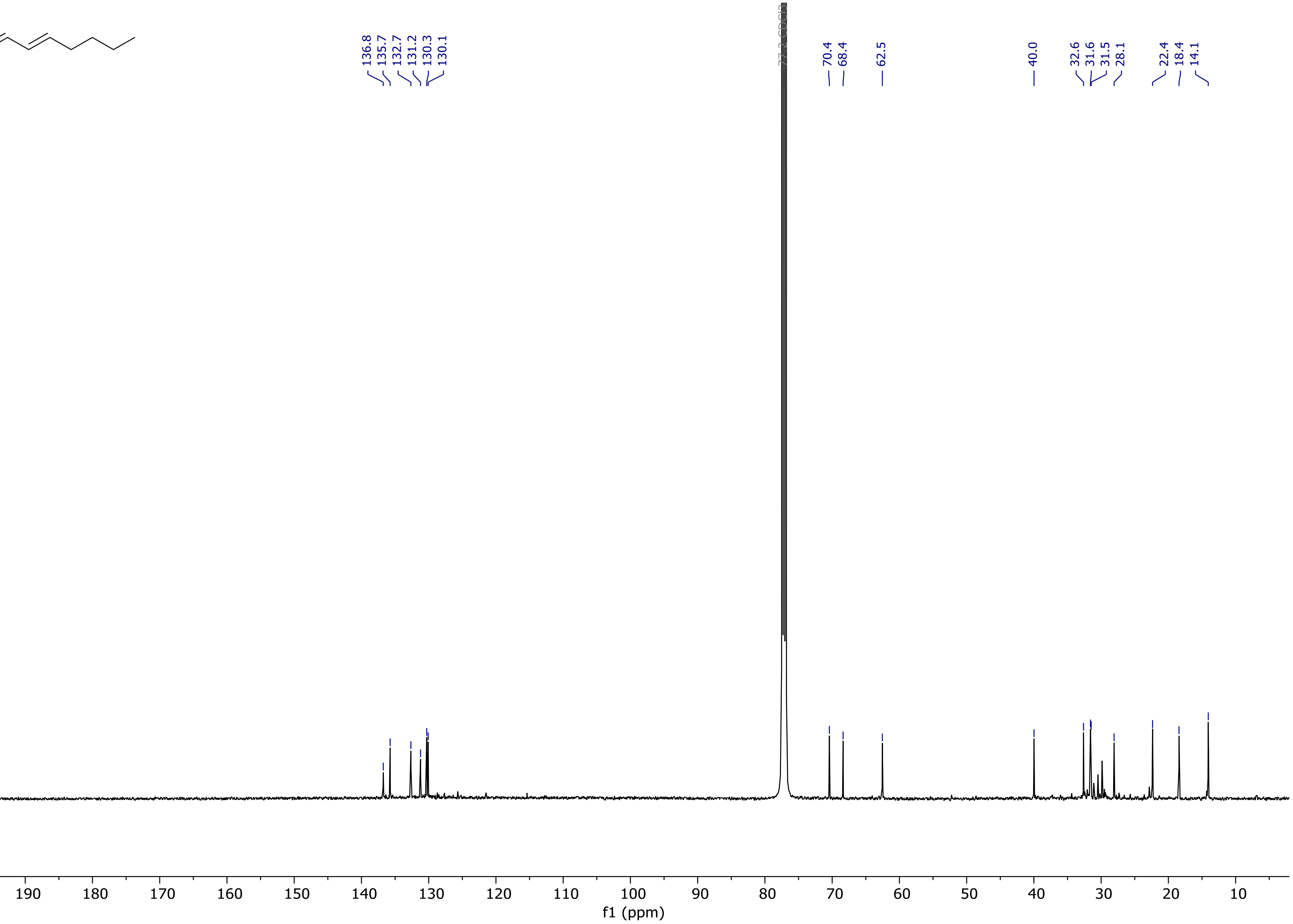

(1*R*,2*S*,3*S*,6*S*,1′*E*,3′*E*,5′*E*)-*N*-Methyl-2-Methyl-6-(deca-1′,3′,5′-trienyl)piperidin-3-ol-*N*-oxide 10 (500 MHz, ^{1}H, $CDCl_3$)

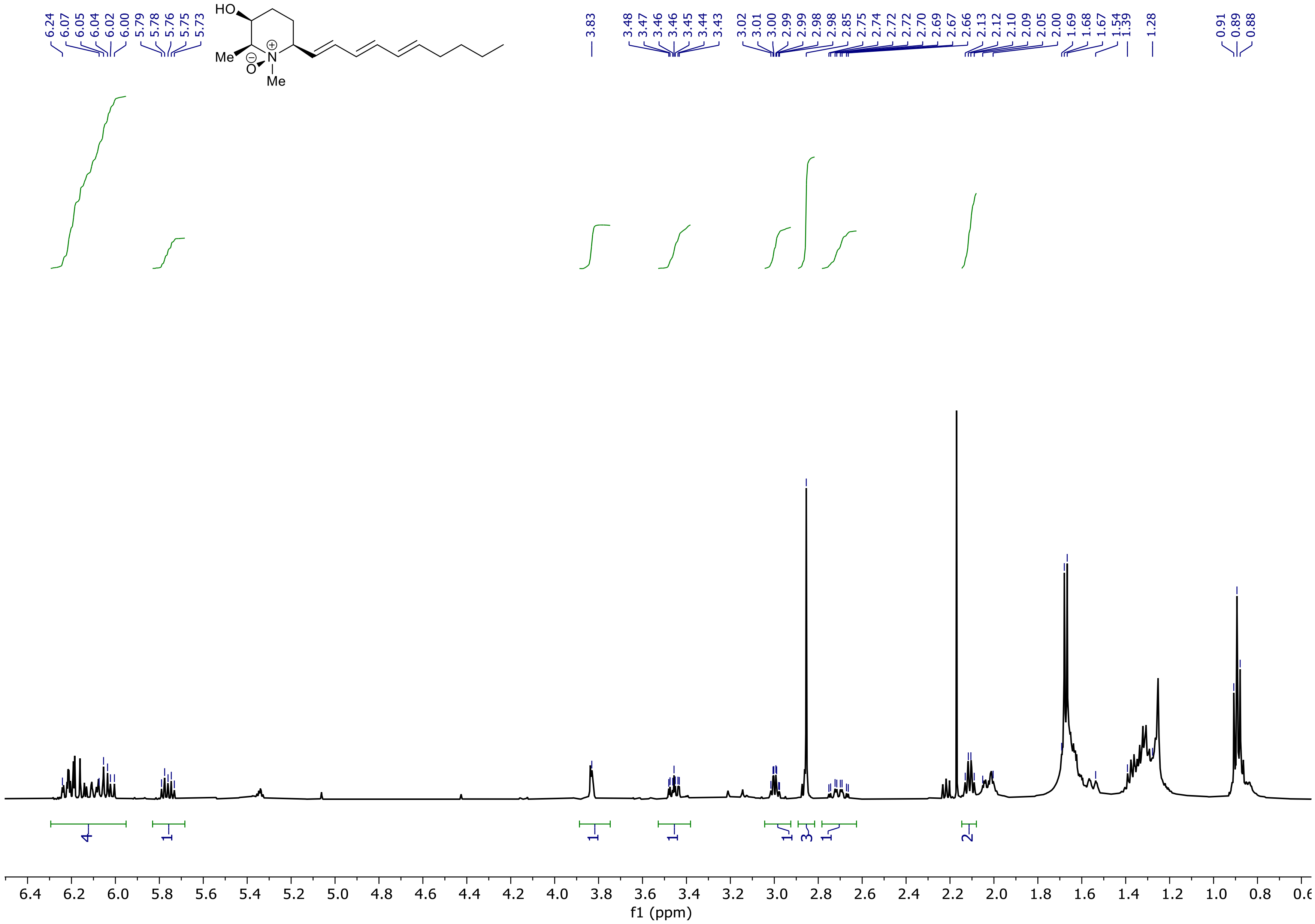

(1*R*,2*S*,3*S*,6*S*,1′*E*,3′*E*,5′*E*)-*N*-Methyl-2-Methyl-6-(deca-1′,3′,5′-trienyl)piperidin-3-ol-*N*-oxide 10 (125 MHz, ^{13}C, $CDCl_3$)

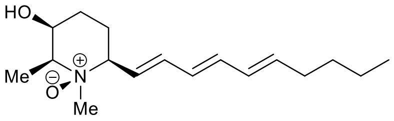

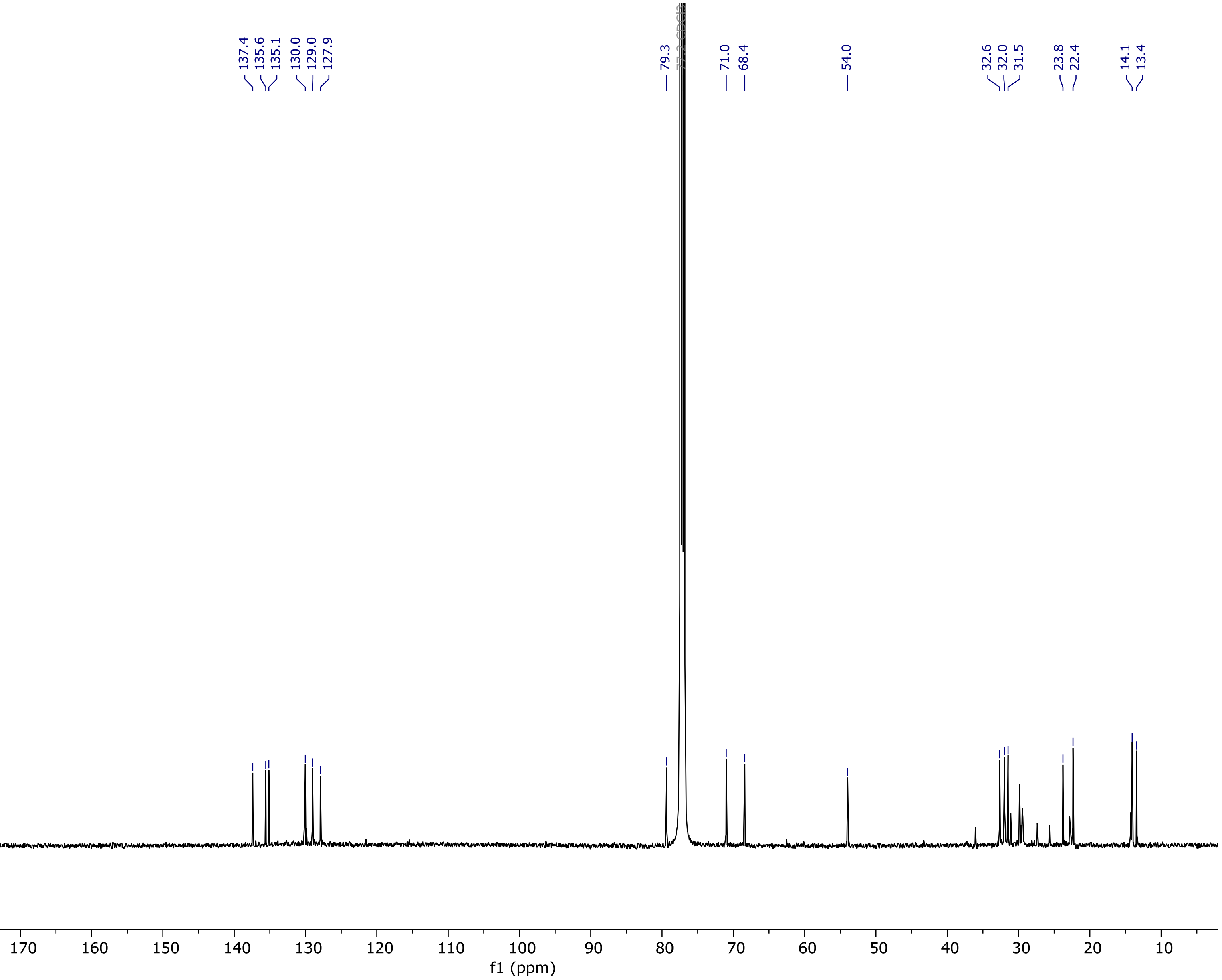

Table SI1: Specific Rotation Values for Synthetic Samples of the Alkaloids and Related Precursors

Compound	Specific Rotation	Compound	Specific Rotation
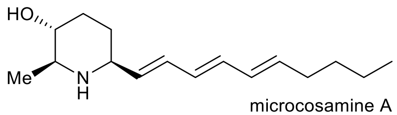 microcosamine A	$[\alpha]_D^{25}$ +2.8 (*c* 0.1 in MeOH)<br>$[\alpha]_D^{25}$ +3.9 (*c* 0.5 in MeOH)<br>$[\alpha]_D^{25}$ +4.2 (*c* 1.0 in MeOH)<br>$[\alpha]_D^{20}$ +4.5 (*c* 1.0 in MeOH)	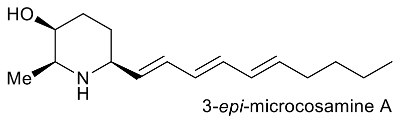 3-*epi*-microcosamine A	$[\alpha]_D^{25}$ +6.2 (*c* 0.1 in $CHCl_3$)
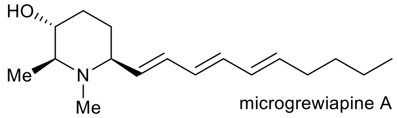 microgrewiapine A	$[\alpha]_D^{25}$ −9.3 (*c* 0.05 in MeOH)<br>$[\alpha]_D^{25}$ −16.0 (*c* 0.1 in MeOH)<br>$[\alpha]_D^{25}$ −24.6 (*c* 0.5 in MeOH)<br>$[\alpha]_D^{25}$ −26.2 (*c* 1.0 in MeOH)	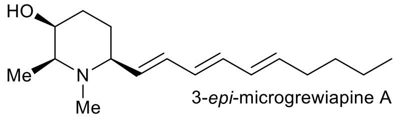 3-*epi*-microgrewiapine A	$[\alpha]_D^{25}$ −15.2 (*c* 1.0 in $CHCl_3$)
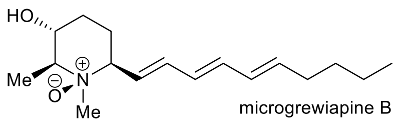 microgrewiapine B	$[\alpha]_D^{25}$ +3.9 (*c* 0.1 in MeOH)<br>$[\alpha]_D^{25}$ +5.3 (*c* 0.5 in MeOH)<br>$[\alpha]_D^{25}$ +6.7 (*c* 1.0 in MeOH)	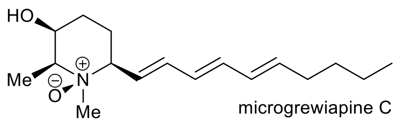 microgrewiapine C	$[\alpha]_D^{25}$ +77.1 (*c* 1.0 in MeOH)
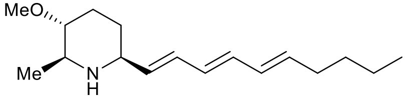	$[\alpha]_D^{25}$ −13.9 (*c* 1.0 in $CHCl_3$)	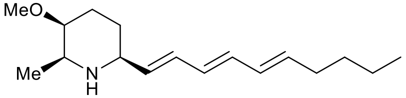	$[\alpha]_D^{25}$ +7.3 (*c* 1.0 in $CHCl_3$)
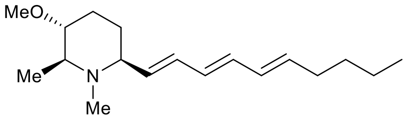	$[\alpha]_D^{25}$ −4.9 (*c* 1.0 in $CHCl_3$)	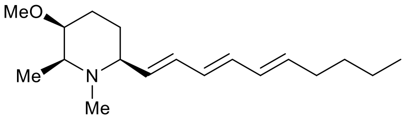	$[\alpha]_D^{15}$ −43.2 (*c* 1.0 in $CHCl_3$)<br>$[\alpha]_D^{22}$ −29.0 (*c* 1.0 in $CHCl_3$)<br>$[\alpha]_D^{22}$ −26.8 (*c* 0.5 in $CHCl_3$)<br>$[\alpha]_D^{22}$ −20.9 (*c* 0.1 in $CHCl_3$)<br>$[\alpha]_D^{22}$ −10.2 (*c* 0.08 in $CHCl_3$)<br>$[\alpha]_D^{22}$ −8.6 (*c* 0.06 in $CHCl_3$)<br>$[\alpha]_D^{22}$ +7.2 (*c* 0.04 in $CHCl_3$)<br>$[\alpha]_D^{24}$ −26.0 (*c* 1.0 in $CHCl_3$)<br>$[\alpha]_D^{25}$ −30.5 (*c* 1.0 in $CHCl_3$)
